# One-pot synthesis of a long-chain metal-backboned polymer

**DOI:** 10.1093/nsr/nwag451

**Published:** 2026-07-23

**Authors:** Zihao Zhao, Fuyao Huang, Yifeng Zhang, Kainan Hong, Chumeng Jiang, Huisheng Peng

**Affiliations:** State Key Laboratory of Molecular Engineering of Polymers, Department of Macromolecular Science, and Institute of Fiber Materials and Devices, Fudan University, Shanghai 200438, China; State Key Laboratory of Molecular Engineering of Polymers, Department of Macromolecular Science, and Institute of Fiber Materials and Devices, Fudan University, Shanghai 200438, China; State Key Laboratory of Molecular Engineering of Polymers, Department of Macromolecular Science, and Institute of Fiber Materials and Devices, Fudan University, Shanghai 200438, China; State Key Laboratory of Molecular Engineering of Polymers, Department of Macromolecular Science, and Institute of Fiber Materials and Devices, Fudan University, Shanghai 200438, China; State Key Laboratory of Molecular Engineering of Polymers, Department of Macromolecular Science, and Institute of Fiber Materials and Devices, Fudan University, Shanghai 200438, China; State Key Laboratory of Molecular Engineering of Polymers, Department of Macromolecular Science, and Institute of Fiber Materials and Devices, Fudan University, Shanghai 200438, China

**Keywords:** metal backbone, gold–gold bond, electron delocalization, polymer, photoluminescence

## Abstract

Metal-backboned polymers, featuring backbones composed exclusively of metal atoms interconnected by consecutive metal–metal bonds, have been proposed as a new class of materials for promising applications in optics, electronics and magnetics; however, they remain unavailable for synthesis. Taking advantage of the long-range aurophilic attraction of gold, we report a straightforward one-pot synthesis method to fabricate metal-backboned polymers. This strategy achieves a degree of polymerization exceeding 50 and stable chain integrity in solution, providing a concise and efficient route for their production. Systematic structural characterizations revealed a one-dimensional Au backbone with continuous Au–Au bonds stabilized by quasi-covalent bonding in synergy with ligand protection. The resulting gold-backboned polymer (GBP) exhibits typical polymer behaviors, including the glass transition temperature and film-forming ability. In addition, GBP exhibits effective electron delocalization, semiconducting properties and long-wavelength phosphorescence, with a preliminary electroluminescent fiber device demonstrating its potential in flexible optoelectronics. This study establishes a general and efficient route for the synthesis of metal-backboned polymers.

## Teaser

A straightforward one-pot synthesis method for long-chain metal-backboned polymers was developed, yielding a GBP with a degree of polymerization exceeding 50 that exhibits typical polymer behaviors, semiconducting properties and potential in flexible optoelectronics.

## INTRODUCTION

A novel class of polymers, metal-backboned polymers, is suggested, with backbones composed exclusively of metal atoms interconnected *via* metal–metal bonds [1,2]. Built as one-dimensional metallic chains at the single-atom level, the unique backbone establishes them as a promising platform for applications in optoelectronic devices, flexible electronics and microwave absorption [3–6]. Thus, many efforts have been made in the recent decade to synthesize these new polymer materials. Unfortunately, although many metal-backboned oligomers have been reported, it remains a challenge to lengthen the metal backbone to realize such polymers. The synthesis of metal-backboned polymers faces two significant challenges. (i) Most metal–metal bonds are too weak to form long polymer chains. (ii) Metal atoms have an intrinsic tendency to form close-packed structures, which favor the formation of two- or three-dimensional aggregates over one-dimensional chains [7–11]. The first controlled synthesis was achieved to extend the length of metal backbones with the use of cage-like multidentate bridging ligands [1]; however, it is difficult to realize a high degree of polymerization because of the increasing miscoordination probability with chain extension.

Herein, we report a strategy that enables metal centers to polymerize directly, achieving spontaneous chain formation and stabilization to produce metal-backboned polymers. Gold is particularly attractive for its long-range aurophilic interactions (up to ∼3.5 Å), which kinetically facilitate the encounter and addition of gold atoms along a growing chain (Fig. 1) [12–18]. Starting from the precursor, the chain growth was driven by chloride abstraction with Ag^+^. Structural insights from single crystals of a chlorine-terminated trinuclear oligomer and mass spectrometry suggest a one-dimensional chain in which gold atoms are alternately bridged by a small-molecule ligand, bis(diphenylphosphino)methane (DPPM) (Fig. 1a). The continuous Au backbone was stabilized by quasi-covalent Au–Au bonding and a protective ligand sheath. Unlike previously reported one-dimensional silver or gold alkynyl chains whose structural integrity depends on crystal packing [19–23], this gold-backboned polymer (GBP) remains intact in solution and exhibits genuine macromolecular behavior, including a defined molecular weight distribution, glass transition temperature and film-forming ability. Furthermore, GBP shows efficient photoluminescence and semiconducting properties, and its potential for use in flexible light-emitting devices is preliminarily explored.

**Figure 1.**
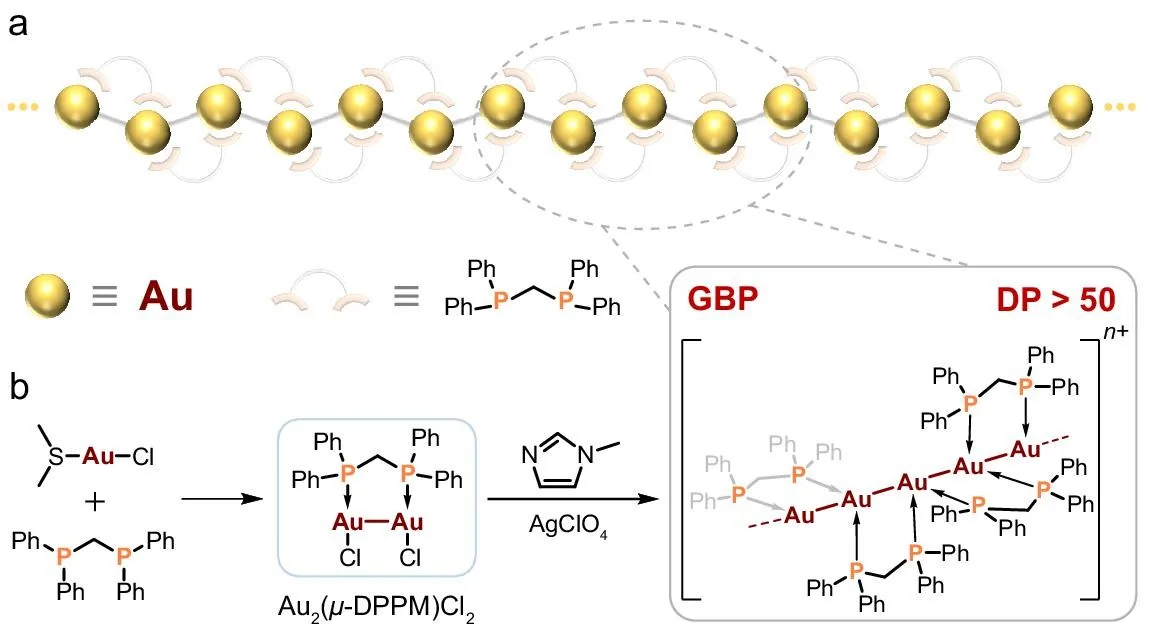
Structure and synthetic route to metal-backboned polymer. (a) Schematic architecture of the molecular structure of GBP. (b) Schematic illustration of the synthetic route. For clarity, counteranions and certain by-products are omitted; a fully balanced equation with charges and by-products is provided in Scheme S1.

## RESULTS AND DISCUSSION

### One-pot synthesis and mechanistic study of GBP

The dinuclear gold precursor Au_2_(μ-DPPM)Cl_2_ was readily obtained by reacting (dimethyl sulfide)gold(I) chloride with DPPM *via* ligand substitution (Fig. 1b and Figs S1–S3). Polymerization was initiated by adding Ag^+^ and *N*-methylimidazole (*N*-MeIm) to a precursor solution. The reaction yielded a polymeric product (Figs S4 and S5) with weight-average molecular weights of up to 34 kDa and polydispersity of 2.15, as determined by gel permeation chromatography coupled with multi-angle laser light scattering (Fig. 2a), using a measured d*n*/d*c* value of 0.1418 mL/g (Fig. S6).

**Figure 2.**
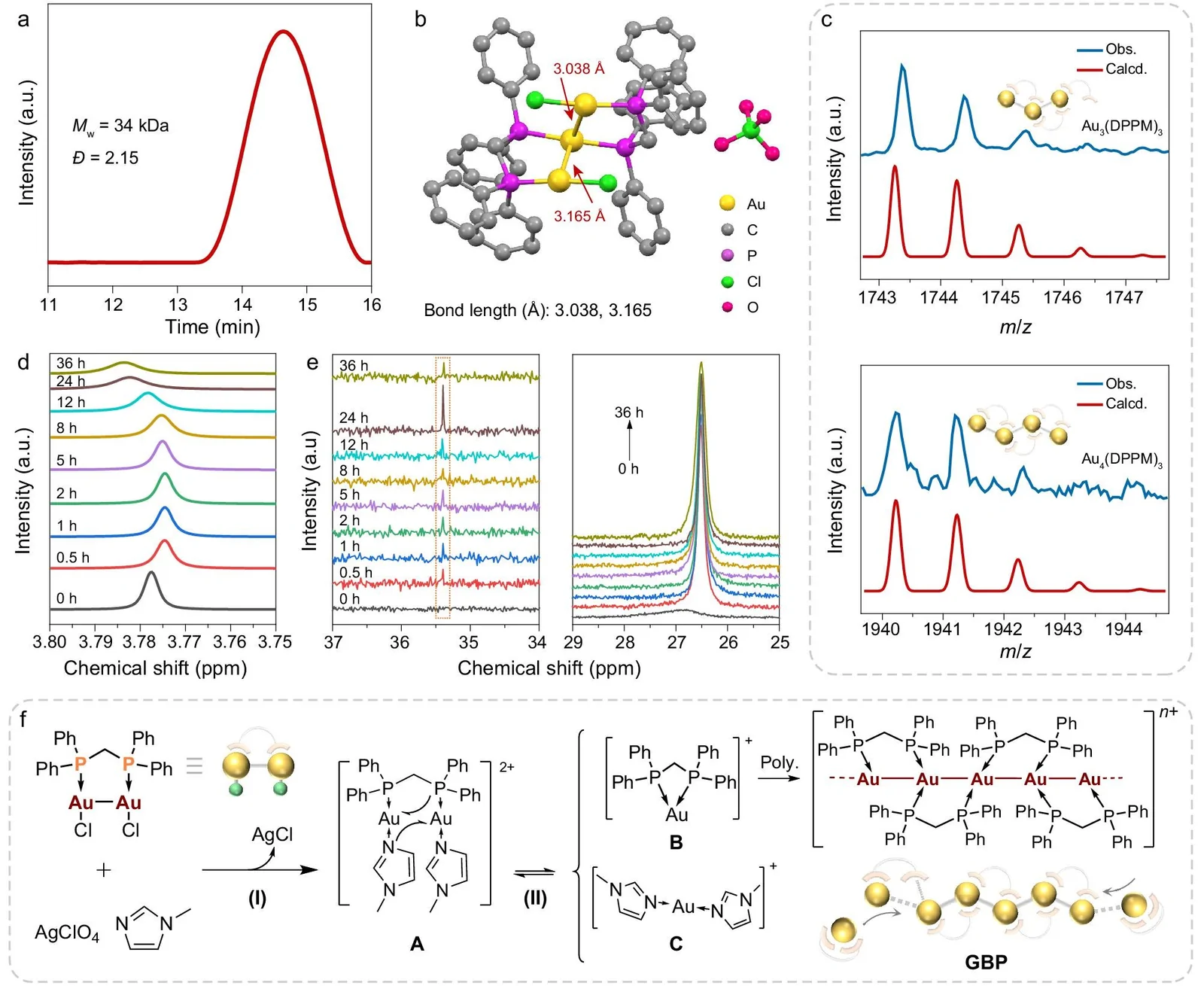
Characterization of the polymerization and structural elucidation of key intermediates. (a) Gel permeation chromatography coupled with multi-angle laser light scattering trace of GBP. (b) Single-crystal structure of the chlorine-terminated trinuclear oligomer [Au_3_(μ-DPPM)_2_Cl_2_]ClO_4_. Hydrogen atoms are omitted for clarity. (c) Magnified matrix-assisted laser desorption/ionization time-of-flight mass spectra (observed, Obs.) and simulated isotope pattern (calculated, Calcd.) of species with the elemental compositions of Au_3_(DPPM)_3_ (top) and Au_4_(DPPM)_3_ (bottom). (d) Selected *in situ*  ^1^H nuclear magnetic resonance spectra (methyl region of *N*-MeIm) recorded at indicated time points after Ag⁺ addition, showing the characteristic two-stage shift of the signal. (e) Selected *in situ*  ^31^P{^1^H} nuclear magnetic resonance spectra recorded at indicated time points, showing the evolution of the signals at 26.89–26.54 ppm and 35.39 ppm. (f) Schematic illustration of the proposed reaction pathway for the polymerization (Poly.). For clarity, counteranions (ClO_4_^−^) and certain by-products are omitted; a full balanced equation is provided in Scheme S1.

The intermediates in the reaction mixture were analyzed to gain insight into the structure of the polymer. By reducing the reaction time and diluting the mixture, we successfully cultured and isolated single crystals of the chlorine-terminated trinuclear oligomer [Au_3_(μ-DPPM)_2_Cl_2_]ClO_4_, whose crystal structure was determined by X-ray diffraction (Fig. 2b and Table S1) [24,25]. The structure revealed three sequentially connected gold atoms with Au–Au distances of 3.038 and 3.165 Å, within the typical range of aurophilicity, and a bond angle of 66.4° (Fig. S7). Adjacent gold atoms were alternately bridged by two DPPM ligands, each coordinating through its two phosphorus atoms to stabilize the Au_3_ core. Notably, the terminal gold atoms retained their chloride ligands, providing reactive sites for subsequent chain extensions. Moreover, by monitoring the reaction mixture with matrix-assisted laser desorption/ionization time-of-flight mass spectrometry, signals with *m*/*z* values consistent with the elemental compositions of Au_3_(DPPM)_3_ and Au_4_(DPPM)_3_ were detected (Fig. 2c), supporting the presence of longer intermediates, in which DPPM likely adopts an alternating bridging pattern similar to that in the trinuclear crystal. Based on these results, we propose a polymer model as a one-dimensional gold chain, in which each two adjacent gold atoms are coordinated by a DPPM ligand in an alternating pattern, with the ligands stabilizing the metal backbone (Fig. 1a). Based on this model, the degree of polymerization was calculated to be 58.

To further assess the thermodynamic feasibility of the proposed chain structure, density functional theory calculations of the dinuclear precursor and chlorine-terminated oligomeric models of increasing length were performed to derive the Gibbs free energy changes for chain extension (Fig. S8). Taking the dinuclear precursor Au_2_(μ-DPPM)Cl_2_ as the zero reference (Δ*G* = 0), we find that the formation of the chlorine-terminated trinuclear species [Au_3_(μ-DPPM)_2_Cl_2_]ClO_4_ is exergonic by −1.99 eV, and further extension to the pentanuclear model releases an additional −6.02 eV. The increasingly negative free energy with increasing chain length demonstrates that chain growth is thermodynamically favored, providing a driving force for polymerization to a high molecular weight.

*In situ* nuclear magnetic resonance spectroscopy was performed to elucidate the polymerization process in detail. Over 24 h, a two-stage shift in ^1^H nuclear magnetic resonance spectrum for the methyl proton signal of *N*-MeIm (∼3.78 ppm) was detected: an initial upfield shift within 0.5 h, followed by a gradual downfield drift and broadening that did not revert to its original position (Fig. 2d and Fig. S9). This behavior indicates that *N*-MeIm participates in polymerization and is ultimately incorporated into other species. Here, the highly reactive [Au_2_(μ-DPPM)]^2+^ dication is generated and immediately coordinated by excess *N*-MeIm to form the stabilized adduct ‘**A**’ (Fig. 2f, Fig. S10 and Note S1) upon rapid chloride abstraction by Ag^+^ [26]. At this stage, the upfield shift of the methyl signal can be attributed to the enhanced magnetic shielding from the adjacent phenyl rings. As the reaction proceeded, the downfield drift signal indicated its release from this shielding environment, implying the dissociation of the *N*-MeIm ligands. It is proposed that the two *N*-MeIm molecules originally bound to ‘**A**’ are transferred to one gold center, forming [Au(*N*-MeIm)_2_]^+^ (intermediate ‘**C**’) and leaving behind the four-membered chelate [Au(DPPM)]^+^ (intermediate ‘**B**’), which then undergoes polymerization *via* Au–Au interactions. This pathway was supported by matrix-assisted laser desorption/ionization time-of-flight mass spectrometry, which captured signals corresponding to ‘**B**’ and ‘**C**’ during the reaction-monitoring process (Fig. S11). Notably, the formation of chelated mononuclear species from dinuclear DPPM-bridged gold(I) precursors has been previously observed [26].

Furthermore, *in situ* proton-decoupled ^31^P{^1^H} nuclear magnetic resonance spectroscopy supported this deduction (Fig. 2e and Fig. S12). At the outset, only a broad weak signal was observed at 26.89 ppm, corresponding to the precursor Au_2_(μ-DPPM)Cl_2_. Within 0.5 h of Ag^+^ addition, this signal shifted upfield to 26.54 ppm and became sharp and intense. This is consistent with chloride removal and the formation of dinuclear DPPM-bridged gold(I) species [26,27]. Concurrently, a weak signal emerged at 35.39 ppm at 0.5 h, and then remained steady during the rapid chain growth phase (0.5–8 h, as determined by gel permeation chromatography, Fig. S13). It increased after 8 h as chain growth slowed and eventually decreased to near baseline by 36 h, when the molecular weight began to decrease, indicating the cessation of chain growth and possible degradation. This signal is tentatively assigned to the chelated mononuclear species ‘**B**’ [28], where the reduced P–Au–P bond angle and ring strain of the four-membered chelate enhance the deshielding of the phosphorus nuclei. Taken together, these observations were consistent with the polymerization pathway shown in Fig. 2f.

### Experimental and theoretical characterization of the Au backbone

Considering the relatively weak nature of conventional aurophilic interactions, we combined experimental and theoretical investigations to probe the formation of the Au backbone and the mechanism underlying its stability. We first employed extended X-ray absorption fine structure and high-resolution transmission electron microscopy to verify the formation of a continuous Au–Au bonded backbone. The Fourier-transformed extended X-ray absorption fine structure (FT-EXAFS) spectrum of GBP exhibits an intense peak at ∼1.68 Å and a weaker one at ∼2.75 Å (Fig. 3a). Wavelet transform analysis was employed to resolve the origin (Fig. 3b and Fig. S14). The peak at 1.68 Å maximizes at low *k* (4–6 Å^–1^), which is characteristic of backscattering from light atoms and is therefore assigned to Au–P coordination from the peripheral DPPM ligands. The peak at 2.75 Å maximizes at high *k* (7–9 Å^–1^), which is typical of heavy-atom backscattering and can be assigned to Au–Au bonds [29]. The Au–Au bonds measured in GBP were shorter than those in [Au_3_(μ-DPPM)_2_Cl_2_]ClO_4_, implying a shortening tendency with chain growth. Notably, GBP shows Au–Au and Au–P bonding signals with coordination numbers of 2.1 and 1.9, respectively (Table S2), which is consistent with the one-dimensional chain model where each gold atom is connected to two neighboring gold atoms and two phosphorus atoms (Fig. 1). These results confirm the formation of an Au backbone with continuous Au–Au bonds.

**Figure 3.**
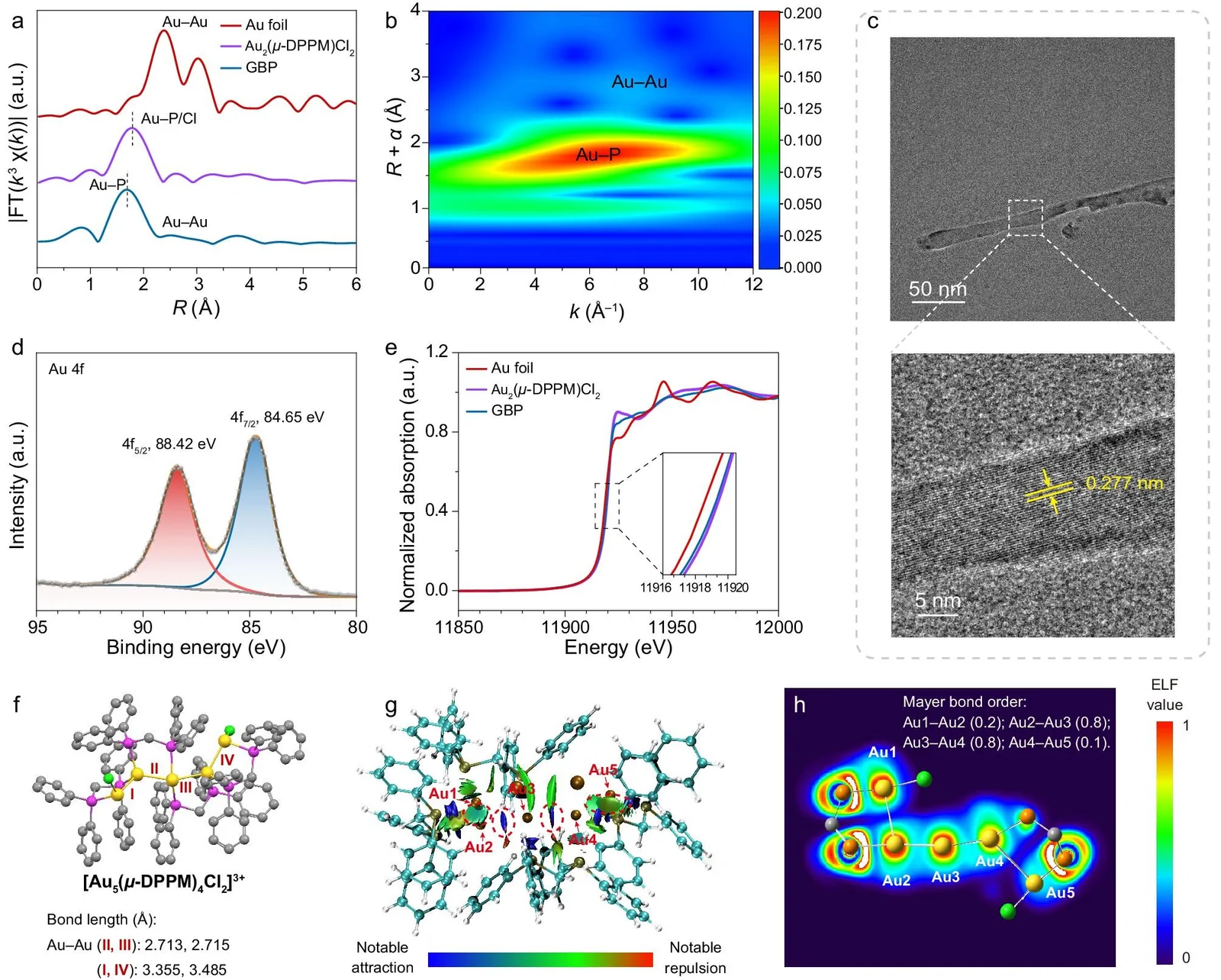
Experimental and theoretical characterization of the GBP structure. (a) Au *L*_3_-edge FT-EXAFS spectra of GBP. (b) Wavelet transform contour of extended X-ray absorption fine structure signal. (c) High-resolution transmission electron microscopy images of GBP and zoomed view of the part of the image denoted by the dashed box. (d) High-resolution X-ray photoelectron spectroscopy spectrum (Au 4f region) of GBP. (e) Au *L*_3_-edge X-ray absorption near-edge structure spectra of GBP, together with Au foil and Au_2_(μ-DPPM)Cl_2_ as reference samples. (f) Density functional theory-optimized structure for the chlorine-terminated pentanuclear oligomer [Au_5_(μ-DPPM)_4_Cl_2_](ClO_4_)_3_. For clarity, the counterions are omitted. (g) Interaction region indicator analysis of GBP backbone. (h) Electron localization function analysis and Mayer bond orders for the indicated Au–Au interactions.

Moreover, the solid-state morphology of the polymer was directly examined using high-resolution transmission electron microscopy. As shown in Fig. 3c, the polymer bundles tended to self-assemble into well-defined rod-like structures, suggesting a one-dimensional chain architecture. The high-resolution image displays a single set of lattice fringes within these rods (Fig. 3c, bottom), with a measured spacing of 0.277 nm. This value agrees well with the Au–Au distances obtained from the extended X-ray absorption fine structure analysis (∼2.75 Å), confirming that the fringes originate from the periodic arrangement of gold atoms along the chain. The presence of only one set of fringes demonstrates the uniform orientation of the polymer chains within the rods, further corroborating the one-dimensional nature of GBP. These real-space observations support the formation of a continuous Au–Au-bonded backbone, confirming the successful synthesis of GBP. Energy-dispersive X-ray spectroscopy mapping performed during transmission electron microscopy imaging (Fig. S15) confirmed the presence of Au, P, Cl, C and O in the rod-like assemblies, thus supporting the compositional model. The uniform O and Cl signals in the energy-dispersive X-ray spectroscopy mapping imply that ClO_4_^−^ counterions are homogeneously dispersed around the Au backbone, likely serving to balance the positive charges and contribute to chain stability.

Furthermore, X-ray photoelectron spectroscopy and X-ray absorption fine structure measurements were performed to probe the electronic structure of GBP. The X-ray photoelectron spectroscopy survey scan (Fig. S16) confirmed the presence of Au, P, Cl, C and O in the GBP. The high-resolution spectrum in the Au 4f region shows a symmetric doublet (Fig. 3d), with binding energies of 84.65 eV (Au 4f_7/2_) and 88.42 eV (Au 4f_5/2_). This symmetric feature, distinct from the asymmetric profile of bulk gold [30], together with the absence of additional Au components, indicates a single chemical environment for all Au atoms, ruling out the coexistence of discrete Au^0^ and Au^+^ domains. The binding energy of Au 4f_7/2_ is shifted by +0.65 eV relative to that of bulk gold (84.0 eV), significantly smaller than the ∼2.0 eV shift typically observed for Au^+^ complexes [31]. This observation suggests a partial positive charge (Au^+δ^) on each Au center rather than a formal Au(I) or Au(0) state. This conclusion is further supported by synchrotron X-ray absorption near-edge structure analysis. The absorption edge of GBP lies between those of the Au foil and Au(I) reference Au_2_(μ-DPPM)Cl_2_, with a smoothly varying profile (Fig. 3e), indicating an average oxidation state between 0 and +1. Together with the homogeneous chemical environment evidenced by X-ray photoelectron spectroscopy, this behavior rules out a physical mixture of Au^0^ and Au^+^ species. Collectively, the X-ray photoelectron spectroscopy and X-ray absorption near-edge structure results reveal a delocalized electronic structure along the one-dimensional Au backbone, in which each Au center carries a uniform partial positive charge (Au^+δ^), with an average oxidation state intermediate between 0 and +1. This observation suggests that the Au–Au interaction in the backbone is not merely aurophilic, but involves appreciable covalent character [15], which substantially enhances the bonding strength, providing an electronic basis for chain stability.

To gain a deeper insight into the bonding nature of the Au backbone, we performed density functional theory calculations on a chlorine-terminated pentanuclear oligomer model, [Au_5_(μ-DPPM)_4_Cl_2_](ClO_4_)_3_ (Fig. 3f and Figs S17 and S18). The optimized structure shows five sequentially connected gold atoms encased by the DPPM ligands. The three central Au atoms (Au2, Au3 and Au4) adopt a nearly linear arrangement (bond angle: 167.27°) with short Au–Au distances of 2.713 and 2.715 Å, indicating strong interactions. In contrast, the terminal Au1–Au2 and Au4–Au5 bonds were significantly elongated (3.355 and 3.485 Å, respectively) and exhibited a bent geometry (Au1–Au2–Au3:102.35°, Au3–Au4–Au5:123.16°), similar to that observed in [Au_3_(μ-DPPM)_2_Cl_2_]ClO_4_. This terminal distortion can be ascribed to steric hindrance from the phenyl rings of the DPPM ligands.

Quantitative analysis of the interaction region indicator corroborates this picture [32]. Strong attractive interactions are observed between Au2, Au3 and Au4 (circled dark blue surfaces in Fig. 3g), with Mayer bond orders of 0.8 for both Au2–Au3 and Au3–Au4 (Fig. 3h), indicating a significant quasi-covalent character [15]. In contrast, the terminal Au1–Au2 and Au4–Au5 interactions are weaker (corresponding to circled green surfaces in Fig. 3g), with Mayer bond orders of 0.2 and 0.1, respectively (Fig. 3h), consistent with aurophilicity. Further electron localization function analysis revealed values of 0.4–0.5 for the regions between Au2, Au3 and Au4 (Fig. 3h), demonstrating incipient metallicity and electron delocalization along the Au backbone. These theoretical results suggest that, despite the intrinsically weak character of aurophilicity, a continuous quasi-covalent bonding character can still form in the interior of the Au backbone, with bond lengths shorter than those of conventional aurophilic interactions.

In addition to the strong Au–Au interactions, the exterior DPPM ligands also play a vital role in stabilizing the Au backbone. Interaction region indicator analyses of [Au_3_(μ-DPPM)_2_Cl_2_]ClO_4_ and [Au_5_(μ-DPPM)_4_Cl_2_](ClO_4_)_3_ revealed abundant π–π interactions among the adjacent phenyl rings of the DPPM ligands (Figs S19 and S20). This dense π–π stacking network effectively counteracts the steric repulsion inherent to the bulky phenyl groups, allowing DPPM to pack tightly around the Au backbone and stabilize each other *via* dispersion forces. By forming a protective sheath outside the backbone, this compact ligand layer physically impedes solvent access, thereby protecting the Au–Au bonds from solvent-induced degradation and ensuring the integrity of the metal backbone.

Taken together, the structural and electronic characterizations reveal a unique bonding character. Although the initial polymerization was driven by the long-range but weak aurophilic attraction, the resulting polymer exhibits Au–Au distances as short as 2.7–2.8 Å and a partial oxidation state, indicative of quasi-covalent bonding stronger than simple aurophilic interactions. This quasi-covalent character, which emerges during chain growth, is beneficial for the stability of the Au backbone. The high polymerization degree arises from a synergy of kinetic and thermodynamic factors: long-range aurophilic attraction kinetically guides chain propagation, quasi-covalent bonding thermodynamically stabilizes the backbone and the peripheral ligand sheath provides solution stability.

### Intrinsic thermal and photophysical properties of GBP

Furthermore, we investigated the intrinsic thermal properties of GBP. Thermogravimetric analysis revealed an onset decomposition temperature (*T*_d_) of ∼295°C, with a residual mass of 40.1% at 600°C (Fig. 4a), indicating that thermal stability is sufficient for conventional processing. Differential scanning calorimetry displayed a clear glass transition temperature (*T*_g_) of 61.1°C (Fig. 4b), suggesting a stable state of GBP at room temperature. This moderately low *T*_g_ proves the segmental mobility of this GBP, which is a key distinction from oligomeric or small-molecule counterparts. In addition, GBP showed good solution processing and film-forming ability (Fig. S21).

**Figure 4.**
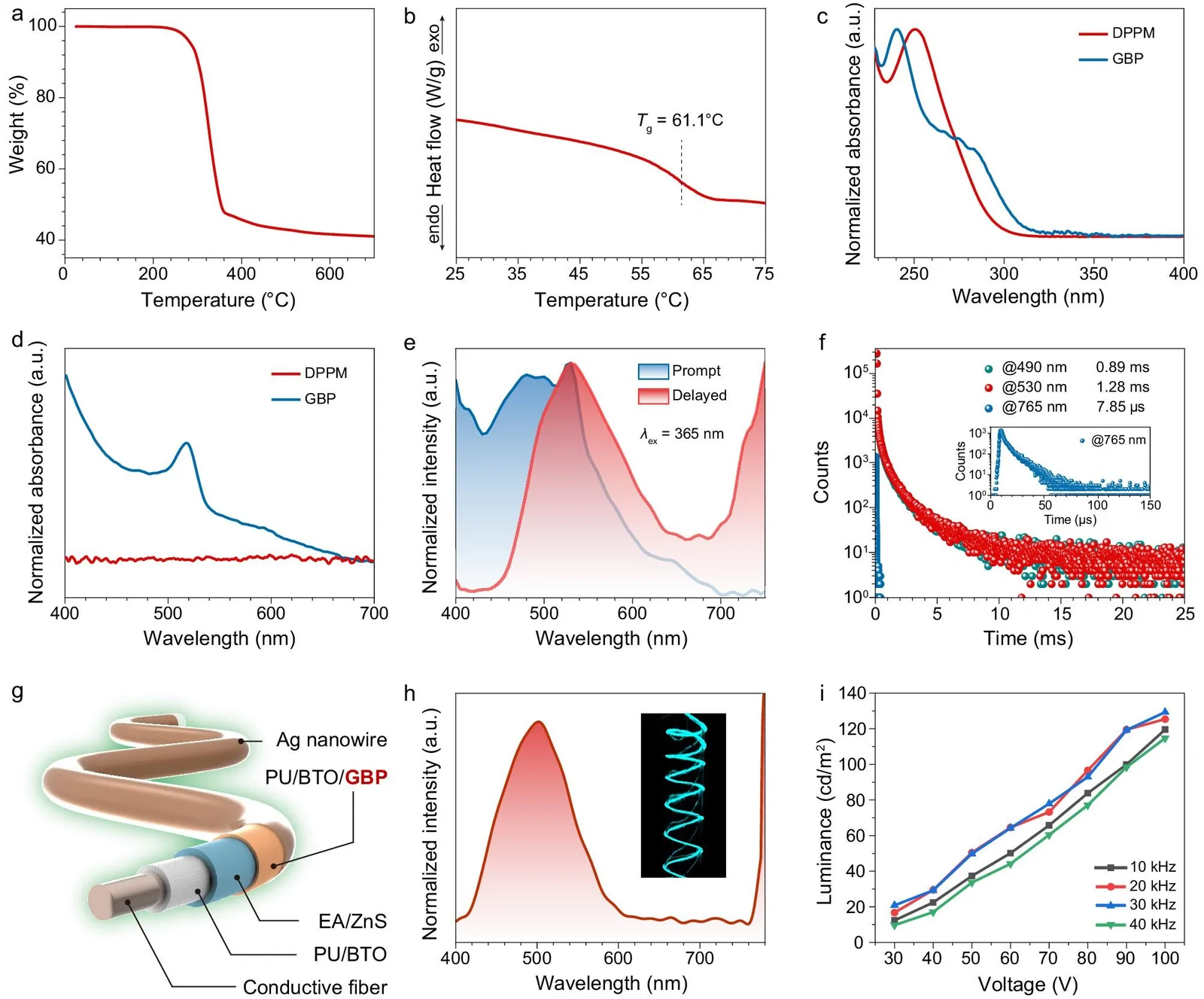
Intrinsic thermal and photophysical properties of GBP. (a) Thermogravimetric analysis and (b) differential scanning calorimetry curves of GBP, showing an onset *T*_d_ of ∼295°C and a *T*_g_ of 61.1°C. (c and d) Absorption spectra of DPPM and GBP. The details in visible region are stressed in panel (d). (e) The prompt and delayed (*t*_d_ = 0.1 ms) emission spectra as well as (f) ms/μs-scale lifetimes of GBP solid powder (λ_ex_ = 365 nm). The time-resolved measurement at 765 nm is zoomed in the inset of panel (f). (g) Schematic illustration of the device structure for the designed alternating-current electroluminescent fiber device. The structure features a ZnS-based emission core, with a GBP layer coated on its surface to serve as the phosphor conversion layer. (h) Emission spectrum of the fiber device. The inset is the luminescent photograph of the fiber device. (i) Luminance of the fiber device at varied voltage and frequency.

To investigate the electronic structure of GBP, the ultraviolet-visible absorption spectra of the polymer and DPPM ligand were determined. The free ligand displayed a single absorption band at ∼250 nm, which was attributed to the π→π* transition of the phenyl rings (Fig. 4c). In contrast, for GBP, the abovementioned peak blue-shifted to ∼240 nm, consistent with the orbital energy level reorganization induced by the electron-withdrawing effect of the coordinated Au centers. Meanwhile, a structured shoulder appeared at ∼275 nm, with an edge extending to 310 nm, together with a weak peak at 515 nm (Fig. 4d). Given their absence in the spectrum of DPPM, they should be assigned to transitions within the one-dimensional Au backbone. Based on the previous electronic structure of one-dimensional gold strings [33], the shoulder can be attributed to an allowed transition that is delocalized along the Au backbone. Its presence provides direct evidence of electron delocalization through continuous Au–Au bonds. The weak peak at 515 nm is ascribed to a forbidden transition originating from the delocalized backbone. Its long-wavelength position reflects the narrowed gap between the highest occupied molecular orbital and the lowest unoccupied molecular orbital resulting from the extended delocalization. Collectively, these spectral features demonstrate the successful formation of continuous Au–Au bonds along the backbone direction and confirm the effective electron delocalization within the Au backbone.

Owing to the strong heavy-atom effect, gold complexes typically exhibit bright phosphorescence in the solid state [34–36]. Therefore, the intrinsic photoluminescence properties of GBP were further investigated. The excitation spectrum shows an optimal excitation wavelength (λ_ex_) of 365 nm (Fig. S22). Under this excitation, the prompt emission spectrum exhibited a main peak at ∼490 nm and a sharp feature at 530 nm, with an emission band extending beyond 700 nm (Fig. 4e). Such emission characteristics give rise to intense cyan photoluminescence with Commission Internationale de l’Éclairage coordinates (0.26, 0.32) (Fig. S23). The delayed emission spectrum revealed two distinct bands (Fig. 4e and Fig. S24): a broad band centered at 530 nm in the 450–650 nm range, corresponding to yellowish-green phosphorescence with coordinates of (0.34, 0.45) (Fig. S23) and an intense near-infrared component beyond 700 nm. Unfortunately, owing to the detection limit of the spectrometer, the maximum of the near-infrared emission could not be precisely determined. Nevertheless, the strong signal in the 700–900 nm region (especially after 850 nm) demonstrates the promising near-infrared phosphorescence of GBP (Fig. S24). Moreover, time-resolved measurements reveal millisecond-scale lifetimes for the visible emission peaks (0.89 and 1.28 ms at 490 and 530 nm, respectively), while a lifetime of 7.85 μs was detected at 765 nm, confirming the triplet nature of these transitions (Fig. 4f). The close correspondence of the emission bands between the prompt and delayed emission spectra supports the strong phosphorescent character of the synthesized GBP.

Electrical measurements revealed that the GBP behaved as a semiconductor. The dark direct-current conductivity of a hot-pressed GBP film was 9.2 × 10^−6^ mS/cm at 25°C. This low film conductivity is consistent with the limited interchain charge hopping expected for a one-dimensional semiconductor. The electronic band gap of GBP, determined by reflection electron energy loss spectroscopy, was found to be 1.83 eV (Fig. S25). Given the polycationic nature of the Au backbone, solution conductivity was also measured. In *N,N*-dimethylformamide (DMF) (2 mg/mL), the conductivity was 134 μS/cm, which is significantly higher than that of pure DMF (18.7 μS/cm), consistent with the ionic nature of the polymer. Similar behavior was observed in dichloromethane (DCM) (2 mg/mL, 311 μS/cm vs 0.18 μS/cm for pure DCM, Fig. S26).

The efficient photoluminescence of GBP suggests its potential use in flexible light-emitting devices. To explore this, we fabricated an alternating-current electroluminescent fiber device by coating a GBP layer onto a ZnS-based alternating-current electroluminescent fiber as the phosphor conversion layer (Fig. 4g). Upon the application of an alternating-current voltage (30–100 V, 10–40 kHz), the device emitted bright cyan light (Fig. 4h, inset). The electroluminescence spectrum (Fig. 4h) consisted of a cyan band centered at ∼500 nm and a strong near-infrared component beyond 750 nm, which closely resembled the delayed emission spectrum of GBP (Fig. 4e). The luminance increased with ascending applied voltage, with a maximum luminance of ∼130 cd/m^2^ (Fig. 4i). This demonstration highlights the potential of GBP for applications in flexible optoelectronic devices.

## CONCLUSION

In conclusion, we have established a straightforward one-pot synthesis strategy to obtain GBP with a polymerization degree exceeding 50, thereby providing a concise and efficient route to metal-backboned polymers. Systematic characterizations confirm a one-dimensional Au backbone, in which Au atoms are interconnected by continuous Au–Au bonds and alternately coordinated by peripheral DPPM ligands. The short Au–Au distances and partial oxidation state indicate a quasi-covalent bonding character, which, in synergy with the kinetic advantage from long-range aurophilic attraction and the protective effect from ligands, leads to such a high degree of polymerization. GBP remains intact in solution and exhibits genuine macromolecular behavior, including a defined molecular weight distribution, glass transition temperature and film-forming ability. Spectroscopic and electronic studies reveal effective electron delocalization along the Au backbone, semiconducting properties and strong phosphorescence. Preliminary application in an electroluminescent fiber device further demonstrates its potential in flexible optoelectronics. This study provides a facile route to metal-backboned polymers, shedding new light on their future development.

## Supplementary Material

nwag451_Supplemental_Files

## Data Availability

Detailed materials and methods can be found in the online Supplementary data.
