# Supplementary material for: One-pot synthesis of a long-chain metal-backboned polymer
Source: Natl Sci Rev. 2026 Jul 23;13(19):nwag451. doi: 10.1093/nsr/nwag451 (PMC13695143; doi:10.1093/nsr/nwag451)

checkCIF/PLATON report

Structure factors have been supplied for datablock(s) 20260114zzh-tir-au-1_auto

THIS REPORT IS FOR GUIDANCE ONLY. IF USED AS PART OF A REVIEW PROCEDURE FOR PUBLICATION, IT SHOULD NOT REPLACE THE EXPERTISE OF AN EXPERIENCED CRYSTALLOGRAPHIC REFEREE.

No syntax errors found.   CIF dictionary   Interpreting this report

Datablock: 20260114zzh-tir-au-1_auto

```
Bond precision:   C-C = 0.0181 A          Wavelength=1.54184

Cell:            a=16.1462(4)       b=18.7091(4)       c=19.3138(6)
                 alpha=84.698(2)    beta=65.381(3)     gamma=68.894(2)
Temperature:     100 K

                  Calculated                   Reported
Volume            4936.7(3)                    4936.8(3)
Space group       P -1                         P -1
Hall group        -P 1                         -P 1
Moiety formula    C50 H44 Au3 Cl2 P4, Cl O4    C50 H44 Au3 Cl2 P4, Cl O4
Sum formula       C50 H44 Au3 Cl3 O4 P4        C50 H44 Au3 Cl3 N0 O4 P4
Mr                1529.99                      1529.98
Dx,g cm-3         2.059                        2.059
Z                 4                            4
Mu (mm-1)         19.501                       19.501
F000              2896.0                       2896.0
F000'             2857.71
h,k,lmax          20,23,24                     20,22,24
Nref              20568                        19500
Tmin,Tmax         0.124,0.142                  0.638,1.000
Tmin'             0.034

Correction method= # Reported T Limits: Tmin=0.638 Tmax=1.000
AbsCorr = MULTI-SCAN
```

Data completeness= 0.948 Theta(max)= 75.850

R(reflections)= 0.0524(16766) wR2(reflections)= 0.1110(19500)

S = 1.047 Npar= 1106

The following ALERTS were generated. Each ALERT has the format
test-name_ALERT_alert-type_alert-level.
Click on the hyperlinks for more details of the test.

Alert level B

```
PLAT213_ALERT_2_B Atom C59            has ADP max/min Ratio .....       4.8 prolat
PLAT213_ALERT_2_B Atom C60            has ADP max/min Ratio .....       4.3 prolat
PLAT213_ALERT_2_B Atom C61            has ADP max/min Ratio .....       4.4 prolat
PLAT213_ALERT_2_B Atom C63            has ADP max/min Ratio .....       4.8 prolat
PLAT213_ALERT_2_B Atom C65            has ADP max/min Ratio .....       4.4 prolat
PLAT213_ALERT_2_B Atom C69            has ADP max/min Ratio .....       4.9 prolat
PLAT213_ALERT_2_B Atom C74            has ADP max/min Ratio .....       4.1 prolat
PLAT213_ALERT_2_B Atom C75            has ADP max/min Ratio .....       4.9 prolat
PLAT220_ALERT_2_B NonSolvent   Resd 1  C   Ueq(max)/Ueq(min) Range      6.9 Ratio
PLAT220_ALERT_2_B NonSolvent   Resd 2  C   Ueq(max)/Ueq(min) Range      7.9 Ratio
PLAT241_ALERT_2_B High   'MainMol' Ueq as Compared to Neighbors of      C38 Check
PLAT241_ALERT_2_B High   'MainMol' Ueq as Compared to Neighbors of      C65 Check
PLAT241_ALERT_2_B High   'MainMol' Ueq as Compared to Neighbors of      C66 Check
PLAT241_ALERT_2_B High   'MainMol' Ueq as Compared to Neighbors of      C68 Check
PLAT241_ALERT_2_B High   'MainMol' Ueq as Compared to Neighbors of      C75 Check
PLAT242_ALERT_2_B Low    'MainMol' Ueq as Compared to Neighbors of      C22 Check
PLAT242_ALERT_2_B Low    'MainMol' Ueq as Compared to Neighbors of      C44 Check
PLAT242_ALERT_2_B Low    'MainMol' Ueq as Compared to Neighbors of      C92 Check
PLAT331_ALERT_2_B Small Aver Phenyl C-C Dist C73      --C78       .    1.36 Ang.
PLAT971_ALERT_2_B Check Calcd Resid. Dens.  1.36Ang From C37           3.03 eA-3
PLAT971_ALERT_2_B Check Calcd Resid. Dens.  1.45Ang From C22           2.96 eA-3
PLAT971_ALERT_2_B Check Calcd Resid. Dens.  1.14Ang From C24           2.85 eA-3
PLAT971_ALERT_2_B Check Calcd Resid. Dens.  1.41Ang From C69           2.73 eA-3
PLAT971_ALERT_2_B Check Calcd Resid. Dens.  1.11Ang From C35           2.63 eA-3
PLAT972_ALERT_2_B Check Calcd Resid. Dens.  0.97Ang From Au6          -2.83 eA-3
```

Alert level C

```
PLAT041_ALERT_1_C Calc. and Reported SumFormula    Strings  Differ     Please Check
              Calc: C50 H44 Au3 Cl3 O4 P4
              Rep.: C50 H44 Au3 Cl3 N0 O4 P4
PLAT213_ALERT_2_C Atom C38            has ADP max/min Ratio .....       3.7 prolat
PLAT213_ALERT_2_C Atom C62            has ADP max/min Ratio .....       3.8 prolat
PLAT213_ALERT_2_C Atom C66            has ADP max/min Ratio .....       3.8 prolat
PLAT213_ALERT_2_C Atom C68            has ADP max/min Ratio .....       3.1 prolat
PLAT213_ALERT_2_C Atom C70            has ADP max/min Ratio .....       3.8 prolat
PLAT213_ALERT_2_C Atom C71            has ADP max/min Ratio .....       4.0 prolat
PLAT213_ALERT_2_C Atom C72            has ADP max/min Ratio .....       3.1 prolat
PLAT213_ALERT_2_C Atom C81            has ADP max/min Ratio .....       3.4 prolat
PLAT213_ALERT_2_C Atom C96            has ADP max/min Ratio .....       3.3 prolat
PLAT214_ALERT_2_C Atom O6       (Anion/Solvent) ADP max/min Ratio       4.1 prolat
PLAT214_ALERT_2_C Atom O8       (Anion/Solvent) ADP max/min Ratio       4.3 prolat
```

```
PLAT222_ALERT_3_C NonSolvent Resd 1  H   Uiso(max)/Uiso(min) Range        6.6 Ratio
PLAT222_ALERT_3_C NonSolvent Resd 2  H   Uiso(max)/Uiso(min) Range        7.9 Ratio
PLAT230_ALERT_2_C Hirshfeld Test Diff for    C44      --C65      .        6.4 s.u.
PLAT230_ALERT_2_C Hirshfeld Test Diff for    C68      --C92      .        5.5 s.u.
PLAT234_ALERT_4_C Large Hirshfeld Difference C22      --C24      .       0.25 Ang.
PLAT234_ALERT_4_C Large Hirshfeld Difference C42      --C72      .       0.23 Ang.
PLAT234_ALERT_4_C Large Hirshfeld Difference C58      --C63      .       0.19 Ang.
PLAT234_ALERT_4_C Large Hirshfeld Difference C60      --C61      .       0.24 Ang.
PLAT234_ALERT_4_C Large Hirshfeld Difference C66      --C92      .       0.25 Ang.
PLAT234_ALERT_4_C Large Hirshfeld Difference C67      --C70      .       0.19 Ang.
PLAT241_ALERT_2_C High   'MainMol' Ueq as Compared to Neighbors of        C24 Check
PLAT241_ALERT_2_C High   'MainMol' Ueq as Compared to Neighbors of        C27 Check
PLAT241_ALERT_2_C High   'MainMol' Ueq as Compared to Neighbors of        C37 Check
PLAT241_ALERT_2_C High   'MainMol' Ueq as Compared to Neighbors of        C39 Check
PLAT241_ALERT_2_C High   'MainMol' Ueq as Compared to Neighbors of        C59 Check
PLAT241_ALERT_2_C High   'MainMol' Ueq as Compared to Neighbors of        C60 Check
PLAT241_ALERT_2_C High   'MainMol' Ueq as Compared to Neighbors of        C69 Check
PLAT241_ALERT_2_C High   'MainMol' Ueq as Compared to Neighbors of        C70 Check
PLAT241_ALERT_2_C High   'MainMol' Ueq as Compared to Neighbors of        C71 Check
PLAT241_ALERT_2_C High   'MainMol' Ueq as Compared to Neighbors of        C72 Check
PLAT241_ALERT_2_C High   'MainMol' Ueq as Compared to Neighbors of        C74 Check
PLAT241_ALERT_2_C High   'MainMol' Ueq as Compared to Neighbors of        C81 Check
PLAT242_ALERT_2_C Low    'MainMol' Ueq as Compared to Neighbors of        C25 Check
PLAT242_ALERT_2_C Low    'MainMol' Ueq as Compared to Neighbors of        C28 Check
PLAT242_ALERT_2_C Low    'MainMol' Ueq as Compared to Neighbors of        C35 Check
PLAT242_ALERT_2_C Low    'MainMol' Ueq as Compared to Neighbors of        C42 Check
PLAT242_ALERT_2_C Low    'MainMol' Ueq as Compared to Neighbors of        C58 Check
PLAT242_ALERT_2_C Low    'MainMol' Ueq as Compared to Neighbors of        C67 Check
PLAT242_ALERT_2_C Low    'MainMol' Ueq as Compared to Neighbors of        C73 Check
PLAT242_ALERT_2_C Low    'MainMol' Ueq as Compared to Neighbors of        C76 Check
PLAT242_ALERT_2_C Low    'MainMol' Ueq as Compared to Neighbors of        C79 Check
PLAT242_ALERT_2_C Low    'MainMol' Ueq as Compared to Neighbors of        C82 Check
PLAT250_ALERT_2_C Large U3/U1 Ratio for <U(i,j)> Tensor(Resd    2)        2.1 Note
PLAT260_ALERT_2_C Large Average Ueq of Residue Including       Cl6      0.107 Check
PLAT331_ALERT_2_C Small Aver Phenyl C-C Dist C79      --C84      .       1.37 Ang.
PLAT342_ALERT_3_C Low Bond Precision on  C-C Bonds ...............    0.01805 Ang.
PLAT411_ALERT_2_C Short Inter H...H Contact  H59      ..H65      .       2.01 Ang.
                                        -x,2-y,1-z  =      2_576 Check
PLAT601_ALERT_2_C Unit-Cell Contains Solvent Accessible VOIDS .LE.         59 Ang**3
PLAT906_ALERT_3_C Large K Value in the Analysis of Variance ......      6.102 Check
PLAT911_ALERT_3_C Missing FCF Refl Between Thmin & STh/L=    0.600        300 Report
              -16  1  0,   5 21  0,   6 21  0,   7 21  0,   8 21  0,   9 21  0,
               10 21  0,  11 21  0,   4 22  0,   5 22  0,   6 22  0,   7 22  0,
                8 22  0,   9 22  0,  -8-22  1,  -7-22  1,  -6-22  1,  -5-22  1,
               -4-22  1, -10-21  1,  -9-21  1,  -8-21  1,  -7-21  1,  -6-21  1,
               -5-21  1,  -4-21  1,  -3-21  1,  -9-20  1,  -8-20  1,  -7-20  1,
                   (  270 More Missing: see the .ckf listing file)
PLAT971_ALERT_2_C Check Calcd Resid. Dens.  1.52Ang From C68           2.43 eA-3
PLAT971_ALERT_2_C Check Calcd Resid. Dens.  1.22Ang From C65           2.17 eA-3
PLAT971_ALERT_2_C Check Calcd Resid. Dens.  1.31Ang From C66           1.75 eA-3
PLAT971_ALERT_2_C Check Calcd Resid. Dens.  1.37Ang From C71           1.72 eA-3
PLAT971_ALERT_2_C Check Calcd Resid. Dens.  0.04Ang From C68           1.67 eA-3
PLAT971_ALERT_2_C Check Calcd Resid. Dens.  0.55Ang From C61           1.64 eA-3
PLAT971_ALERT_2_C Check Calcd Resid. Dens.  0.65Ang From Au6           1.55 eA-3
PLAT971_ALERT_2_C Check Calcd Resid. Dens.  0.47Ang From C69           1.52 eA-3
PLAT972_ALERT_2_C Check Calcd Resid. Dens.  0.49Ang From C69          -2.11 eA-3
PLAT972_ALERT_2_C Check Calcd Resid. Dens.  0.81Ang From Au2          -1.59 eA-3
```

```
PLAT972_ALERT_2_C Check Calcd Resid. Dens.  0.30Ang From C71           -1.59 eA-3
PLAT972_ALERT_2_C Check Calcd Resid. Dens.  0.61Ang From C68           -1.55 eA-3
PLAT977_ALERT_2_C Check Negative Difference Density on H37       .     -0.37 eA-3
PLAT977_ALERT_2_C Check Negative Difference Density on H38       .     -0.41 eA-3
PLAT977_ALERT_2_C Check Negative Difference Density on H59       .     -0.41 eA-3
PLAT977_ALERT_2_C Check Negative Difference Density on H60       .     -0.42 eA-3
```

Alert level G

```
CELLZ01_ALERT_1_G Difference between formula and atom_site contents detected.
CELLZ01_ALERT_1_G ALERT: Large difference may be due to a
            symmetry error - see SYMMG tests
           From the CIF: _cell_formula_units_Z    4
           From the CIF: _chemical_formula_sum  C50 H44 Au3 Cl3 N0 O4 P4
           TEST: Compare cell contents of formula and atom_site data

           atom    Z*formula  cif sites diff
           C        200.00    200.00    0.00
           H        176.00    176.00    0.00
           Au        12.00     12.00    0.00
           Cl        12.00     12.00    0.00
           N          4.00      0.00    4.00
           O         16.00     16.00    0.00
           P         16.00     16.00    0.00
PLAT003_ALERT_2_G Number of Uiso or U(i,j)  Restrained non-H-Atoms        25 Report
PLAT083_ALERT_2_G SHELXL Second Parameter in WGHT  Unusually Large      90.82 Why ?
PLAT177_ALERT_4_G The CIF-Embedded .res File Contains DELU Records         4 Report
PLAT178_ALERT_4_G The CIF-Embedded .res File Contains SIMU Records         6 Report
PLAT186_ALERT_4_G The CIF-Embedded .res File Contains ISOR Records         2 Report
PLAT192_ALERT_3_G A Non-default DELU Restraint Value for First Par     0.0010 Report
PLAT192_ALERT_3_G A Non-default DELU Restraint Value for SecondPar     0.0010 Report
PLAT192_ALERT_3_G A Non-default DELU Restraint Value for First Par     0.0010 Report
PLAT192_ALERT_3_G A Non-default DELU Restraint Value for SecondPar     0.0010 Report
PLAT192_ALERT_3_G A Non-default DELU Restraint Value for First Par     0.0010 Report
PLAT192_ALERT_3_G A Non-default DELU Restraint Value for SecondPar     0.0010 Report
PLAT192_ALERT_3_G A Non-default DELU Restraint Value for First Par     0.0010 Report
PLAT192_ALERT_3_G A Non-default DELU Restraint Value for SecondPar     0.0010 Report
PLAT244_ALERT_4_G Low    'Solvent' Ueq as Compared to Neighbors of       Cl5 Check
PLAT244_ALERT_4_G Low    'Solvent' Ueq as Compared to Neighbors of       Cl6 Check
PLAT860_ALERT_3_G Number of Least-Squares Restraints .............       160 Note
PLAT912_ALERT_4_G Missing # of FCF Reflections Above STh/L=  0.600       709 Note
PLAT933_ALERT_2_G Number of HKL-OMIT Records in Embedded .res File         8 Note
                4  8  9,   6 -3  7,   6 -1  9,   7 -1 10,   7  0  8,   7  2  6,
                8 -1  9,   9  3  6,
PLAT969_ALERT_5_G The 'Henn et al.' R-Factor-gap value ..........      2.688 Note
              Predicted wR2: Based on SigI**2  4.13 or SHELX Weight 10.61
PLAT978_ALERT_2_G Number C-C Bonds with Positive Residual Density.         1 Info
```

---

0 **ALERT level A** = Most likely a serious problem - resolve or explain
25 **ALERT level B** = A potentially serious problem, consider carefully
68 **ALERT level C** = Check. Ensure it is not caused by an omission or oversight
22 **ALERT level G** = General information/check it is not something unexpected

3 ALERT type 1 CIF construction/syntax error, inconsistent or missing data

```
85 ALERT type 2 Indicator that the structure model may be wrong or deficient
14 ALERT type 3 Indicator that the structure quality may be low
12 ALERT type 4 Improvement, methodology, query or suggestion
 1 ALERT type 5 Informative message, check
```

It is advisable to attempt to resolve as many as possible of the alerts in all categories. Often the minor alerts point to easily fixed oversights, errors and omissions in your CIF or refinement strategy, so attention to these fine details can be worthwhile. It is up to the individual to critically assess their own results and, if necessary, seek expert advice.

PLATON version of 26/09/2025; check.def file version of 20/09/2025

---

duplicate check

No duplication found

Datablock 20260114zzh-tir-au-1_auto - ellipsoid plot

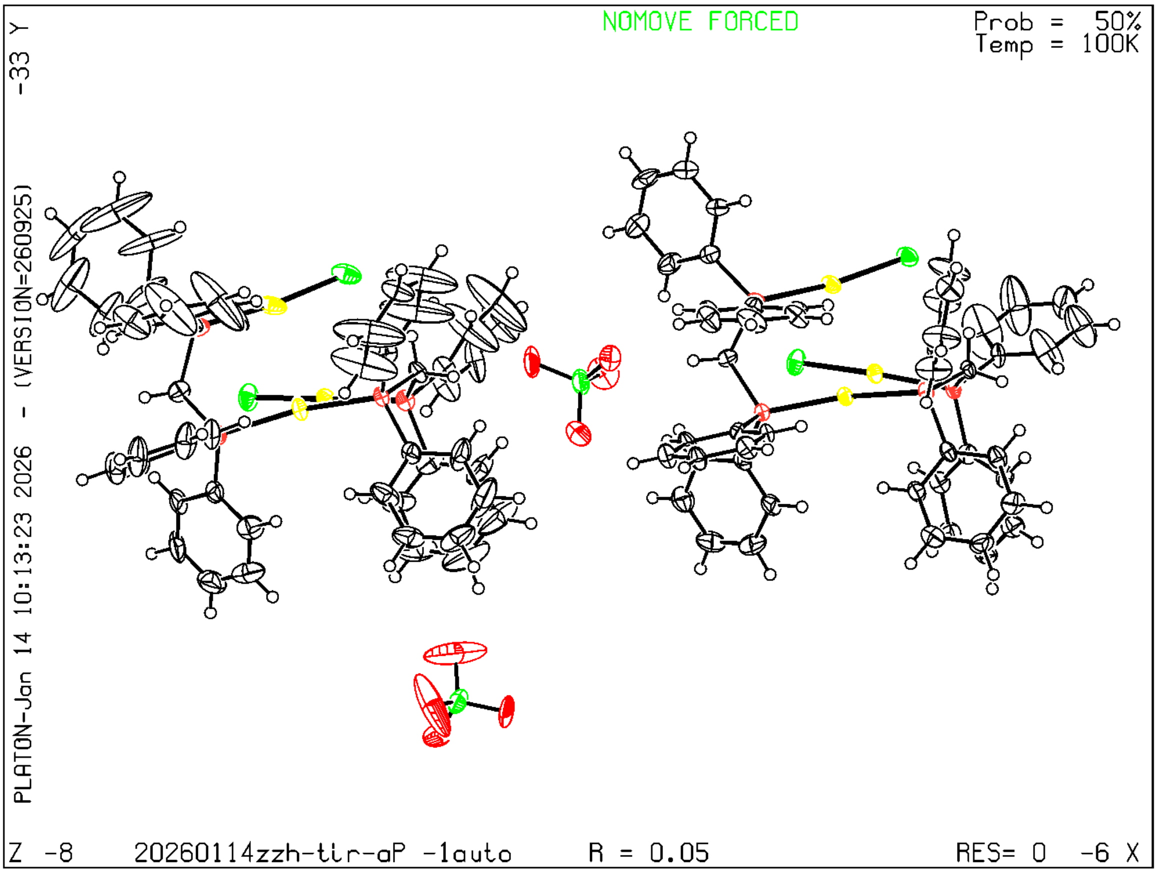

Supplement: Supplementary file 1 [file nwag451_supplemental_files.zip › checkcif for [Au3(u-DPPM)2Cl2]ClO4.pdf]
