# Supplementary material for: One-pot synthesis of a long-chain metal-backboned polymer
Source: Natl Sci Rev. 2026 Jul 23;13(19):nwag451. doi: 10.1093/nsr/nwag451 (PMC13695143; doi:10.1093/nsr/nwag451)
Supplement: Supplementary file 1 [file nwag451_supplemental_files.zip › Supporting information for NSR_MS-2026-1286.R1.pdf]

Supporting Information

One-pot synthesis of a long-chain metal-backboned polymer

Zihao Zhao†, Fuyao Huang†, Yifeng Zhang, Kainan Hong, Chumeng Jiang, Huisheng Peng*

State Key Laboratory of Molecular Engineering of Polymers, Department of Macromolecular Science, and Institute of Fiber Materials and Devices, Fudan University, Shanghai 200438, China.

***Corresponding author**. E-mail: penghs@fudan.edu.cn

†Equally contributed to this work.

1. Experimental

1.1 Reagents and materials

Dimethyl sulfoxide (DMSO-d_6, 99.8%) with 0.03% (v/v) tetramethylsilane as internal standard and chloroform-d ($CDCl_3$,99.8%), was purchased from Cambridge Isotope Laboratories. Trans-2-[3-(4-tert-butylphenyl)-2-methyl-2-propenylidene]malononitrile (DCTB, 98.0%) and sodium trifluoroacetate (98.0%) were purchased from Tokyo Chemical Industry (TCI) Co., Ltd. Anhydrous *N*,*N*-dimethylformamide (DMF, 99.8%) was purchased from Sigma-Aldrich. Silver-plated nylon yarns (100D, Hengtong Xsilver Specialty Textile Co., Ltd) were used as the inner conductive fiber electrode. Waterborne polyurethane (WPU, solid content 50 *wt*.%) was purchased from Shanghai Sisheng Polymer Materials Co., Ltd. (China). $BaTiO_3$ (Shanghai Dianyang Industry Co., Ltd) was used as the dielectric material. ZnS phosphors (Shanghai Keyan Phosphor Technology Co., Ltd) were employed as luminescent materials. Silver nanowire dispersion (AgNWs, 2 mg/mL in ethanol, diameter 30 ± 5 nm, length ~25 μm) was obtained from Suzhou Chenye New Materials Co., Ltd. (China). All other reagents and solvents including bis(diphenylphosphino)methane (> 97.0%), *N*-Methylimidazole (99%), chloro(dimethylsulfide)gold(I) (98%), silver(I) perchlorate ($AgClO_4$, 97%), dichloromethane (> 99.5%), acetone (> 99.5%), diethyl ether (≥ 99.7%), ethyl acetate (99.8%), *n*-hexane (99.7%) were of reagent grade, bought from Adamas Reagent Co., Ltd., and used as received.

1.2 Instrumentation

^1H and ^{31}P nuclear magnetic resonance (NMR) spectra were obtained from a Bruker Avance III HD 600 MHz NMR spectrometer. Matrix-assisted laser desorption/ionization time-of-flight mass spectrometry (MALDI-TOF MS) was measured using an AB SCIEX 5800 mass spectrometer with DCTB as the matrix and sodium trifluoroacetate as the ionization reagent (when necessary).

All gel permeation chromatography (GPC) data were collected *via* an instrument equipped with an isocratic pump (1260 Infinity II, Agilent), a multi-angle static light scattering (MALS) detector with the detection wavelength at 658 nm (DAWN HELEOS-II, Wyatt Technology), and a differential refractometer (dRI) detector (Optilab T-rEX, Wyatt Technology). Separations were performed by serially connected size exclusion columns which were maintained at a temperature of 40 ºC using DMF containing 0.1 M LiBr as the mobile phase. The dn/dc value of the gold-backboned polymer (GBP) was calculated through standard curve collected *via* a dRI detector (Optilab T-rEX, Wyatt Technology). The weight-average molecular weights (M_w)

were determined based on this *d*n/*d*c value by using the internal calibration system processed by the software ASTRA 7 (version 7.1.3.15, Wyatt Technology).

Single-crystal structures were collected on a Bruker D8 VENTURE CMOS Photon II X-ray diffractometer with a Helios MX multilayer monochromator and Cu Ka radiation (λ = 1.54178 Å). Data collection, unit cell refinement and data reduction were performed using APEX3 v2019.11-0. The structure was solved by Intrinsic Phasing method and refined by full-matrix least-squares on F2 with anisotropic displacement parameters for the non-H atoms using SHELXTL program package. The hydrogen atoms on carbon were calculated in ideal positions with isotropic displacement parameters set to 1.2×Ueq of the attached atom (1.5×Ueq for methyl hydrogen atoms). The hydrogen atoms bound to nitrogen were located in a ΔF map and refined with isotropic displacement parameters.

X-ray photoelectron spectroscopy (XPS) was carried out on a PHI Genesis 500 with a monochromatic Al-Kα X-ray source (hv = 1486.7 eV). The detection was performed perpendicularly to the sample surface with a spot size of 200 μm using a constant energy analyzer mode. XPS spectra were recorded with a 0.1 eV energy step size. The peaks were quantified on the basis of Wagner atomic sensitivity factor.

Reflected electron energy loss spectroscopy (REELS) was measured on a PHI Genesis 500 using a primary electron beam of approximately 1 keV in high energy mode, with a beam spot size of around 1 mm. The REELS measurement involved directing a constant high-energy electron beam onto the sample surface and analyzing the energy of the electrons reflected or backscattered by the surface. The obtained REELS spectra consisted of both elastically and inelastically backscattered electrons. The intensity of elastically scattered electrons typically exceeded that of inelastically scattered electrons, creating a prominent energy loss peak at zero loss energy, known as elastic peak.

Au L_3-edge analysis was performed with Si(111) crystal monochromators at the BL11B beamline at the Shanghai Synchrotron Radiation Facility (SSRF) (Shanghai, China). Before the analysis at the beamline, samples were pressed into thin sheets with 1 cm in diameter and sealed using Kapton tape film. The X-ray absorption fine structure (XAFS) spectra were recorded at room temperature using a 4-channel Silicon Drift Detector (SDD) Bruker 5040. Au L_3-edge extended X-ray absorption fine structure (EXAFS) spectra were recorded in transmission mode. Negligible changes in the line-shape and peak position of Au L_3-edge X-ray absorption near edge structure (XANES) spectra were observed between two scans taken for a specific sample. The XAFS spectra of standard samples [Au foil and Au_2(μ-DPPM)Cl_2] were recorded in

transmission mode. The spectra were processed and analyzed by the software codes Athena and Artemis.

Microstructural characterization was carried out on a Thermo Fisher Spectra 300 transmission electron microscope operating at 300 kV. High-resolution transmission electron microscopy (HRTEM) imaging and selected-area electron diffraction were complemented by energy-dispersive X-ray spectroscopy (EDS) mapping. The integrated EDS system, possessing an energy resolution of 136 eV, was employed in scanning transmission electron microscopy (STEM) mode with a sub-ångström probe size of 65 pm to ensure high spatial resolution.

Ultraviolet-visible (UV-vis) absorption spectra were recorded on a Perkin-Elmer Lambda 750 spectrophotometer. Prompt and delayed emission spectra, as well as lifetimes were measured on an Edinburgh FLS1000 photoluminescence spectrometer.

Thermogravimetric analysis (TGA) was carried out on a Mettler-Toledo TGA 1 and SDT Q600 TG-DTA analyzer under N_2 atmosphere, with a temperature range from 30 to 800 °C and a ramp rate of 10 °C min^{-1}. Prior to the TGA, the samples were activated at 100 °C for 30 minutes to remove any moisture.

Differential scanning calorimetry (DSC) measurements were carried out on a TA Instruments Q2000 under N_2 atmosphere, with a temperature range from −70 to 80 °C and a ramp rate of 5 °C min^{-1}.

The electrical conductivity was measured using a Hach HQ2100 portable multiparameter meter equipped with a conductivity electrode. The samples included 2 mg/mL GBP in *N*,*N*-dimethylformamide, 2 mg/mL GBP in dichloromethane, and corresponding pure solvents. For each measurement, an aliquot of the sample was transferred into a clean, dry beaker, and the electrode was directly immersed into the solution. The conductivity value was recorded after the reading had stabilized. All measurements were performed at room temperature. The electrode was carefully rinsed with the corresponding pure solvent and dried between measurements to avoid cross-contamination. Each sample was measured in triplicate, and the average value was reported.

The alternating-current electroluminescent fiber device was excited with a functional waveform generator (3300B, Keysight) and a high-voltage amplifier (ATA-2081, Aigtek). The electroluminescent characteristics (luminance and luminescence spectrum) of the device were measured by a spectroradiometer (PR680, Photoresearch). The photographs were captured by a digital camera (α6000, Sony).

Note: Unless specified, all characterizations were carried out under ambient conditions.

1.3 Computational study

Structure optimization:

Theoretical Calculations were employed by density functional theory (DFT) calculations to optimize the ground state geometries of $[Au_5(\mu\text{-DPPM})_4Cl_2](ClO_4)_3$ using the hybrid B3LYP functional[1,2] and the 6-31g(d) basis set[3]. For gold atoms, the Los Alamos effective core potential basis set (LANL2DZ)[4] was used. All calculations were carried out using the Gaussian 16 program[5]. Meanwhile, interaction region indicator (IRI), Mayer bond orders, and electron localization function (ELF) analysis for $[Au_5(\mu\text{-DPPM})_4Cl_2](ClO_4)_3$ were conducted through Multiwfn package[6] and VMD using the wave functions calculated by Gaussian 16 program.

Gibbs free energy:

All quantum chemical calculations were performed with the ORCA 6.1.0 program package[7,8]. Geometry optimizations and harmonic vibrational frequency calculations were carried out using the composite r²SCAN-3c method[9]. The r²SCAN-3c approach combines the r²SCAN meta-GGA functional with a tailored triple-ζ basis set, D4 London dispersion correction, and geometrical counterpoise correction, and has been recommended as a robust and efficient method for thermochemistry, molecular geometries, non-covalent interactions, and transition-metal complexes. In ORCA, r²SCAN-3c is available for elements up to Lr, making it applicable to the present Au-containing organometallic systems.

Solvent effects were included using the conductor-like polarizable continuum model[10] (CPCM) with dichloromethane as the solvent. Tight SCF convergence criteria were applied throughout the calculations, and the SlowConv option was used to improve the SCF convergence behavior for the Au-containing organometallic species. Harmonic frequency calculations were performed at the same level of theory to confirm the optimized structures as local minima, as indicated by the absence of imaginary frequencies, and to obtain zero-point energy and thermal corrections. Unless otherwise stated, the Gibbs free energies were evaluated at 298.15 K and 1 atm. The relative Gibbs free energies were used to assess the thermodynamic feasibility of Au-chain growth.

1.4 Synthetic and preparation procedures

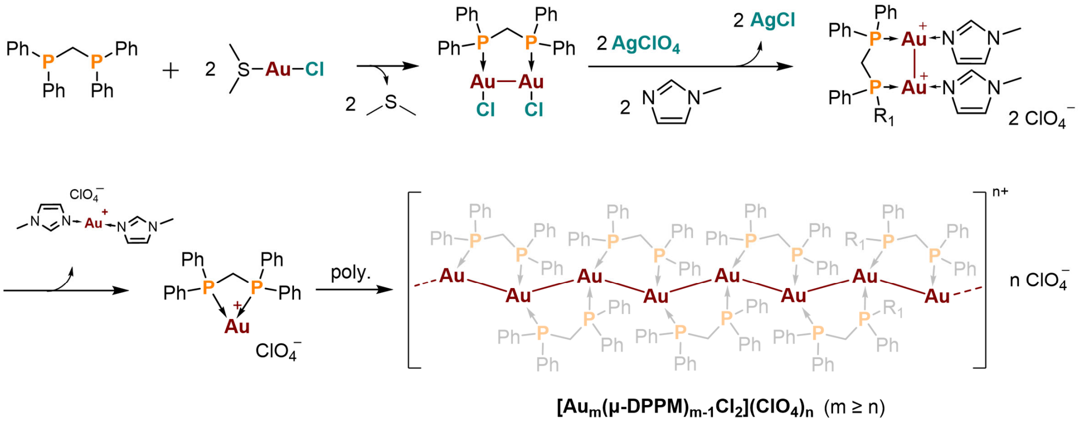

Scheme S1. Total synthetic route of GBP.

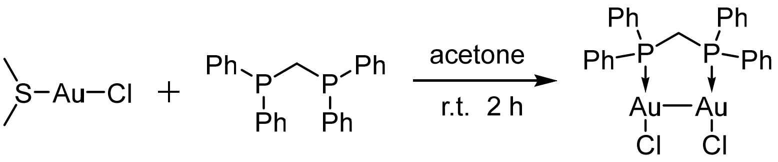

Scheme S2. Synthetic route of Au_2(μ-DPPM)Cl_2.

Synthesis of bis(chlorogold(I)) bis(diphenylphosphino)methane [Au_2(μ-DPPM)Cl_2]. Au_2(μ-DPPM)Cl_2 was synthesized as previously reported.[11] A solution of bis(diphenylphosphino)methane (0.83 g, 2.15 mmol) in acetone (15 mL) was added to a suspension of chloro(dimethylsulfide)gold(I) [AuClS(Me)$_2$] (1.27 g, 4.30 mmol) in acetone (30 mL). The mixture was stirred for 2 h to give a white precipitate, which was collected by filtration, washed with acetone, diethyl ether and *n*-hexane, and further dried under vacuum. Yield: 1.57 g (86%). ^{1}H NMR (600 MHz, DMSO-d_6, ppm): δ 7.77 (q, J = 7.2 Hz, 8H), 7.48 (dt, J = 45.4, 7.9 Hz, 12H), 4.68 (t, J = 12.8 Hz, 2H). ^{31}P NMR (243 MHz, DMSO-d_6, ppm): δ 27.15 (s, PPh_2). MS (MALDI, m/z): [M-Cl]$^+$ calcd for $C_{25}H_{22}Au_2ClP_2$, 813.02; Found, 812.67.

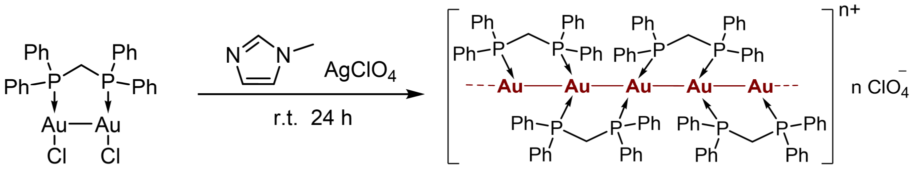

Scheme S3. Synthetic route of GBP.

Synthesis of GBP. Under an argon atmosphere and protected from light, Au_2(μ-DPPM)Cl_2 (500 mg, 0.59 mmol) and $AgClO_4$ (1.22 g, 5.9 mmol) were sequentially added to 50 mL of dry dichloromethane. *N*-Methylimidazole (103 μL) was then added under stirring, and the mixture

was stirred at room temperature for 24 hours. The supernatant was collected by centrifugation and concentrated under reduced pressure. The residue was redissolved in 10 mL of dichloromethane, and 30 mL of cold *n*-hexane was added for reprecipitation. The resulting white solid was collected by centrifugation. This recrystallization process was repeated for 2 to 3 times. The final product was dried under vacuum to afford 293 mg of the GBP. Before GPC characterization, the polymer solution was further filtered through a 0.1 μm membrane (repeated for 2 to 3 times) to remove possible aggregates. ^{1}H NMR (600 MHz, $CDCl_3$, ppm): δ 7.68 (q, J = 7.0 Hz, 8H), 7.42–7.31 (m, 12H), 4.27 (t, J = 12.9 Hz, 2H). ^{31}P NMR (243 MHz, $CDCl_3$, ppm): δ 26.54 (s, PPh_2)

Preparation of alternating-current electroluminescent fiber device. The conductive fiber was employed as the inner electrode, and a dielectric precursor dispersion was prepared by dispersing $BaTiO_3$ particles into 50 *wt*.% waterborne polyurethane (WPU) at a weight ratio of 1:3.5. The conductive fiber was dipped into the dielectric precursor dispersion and pulled out at a rate of 10 m/min through a homemade die (with a diameter of 160 μm) and dried at 80 °C. Commercially sourced ZnS phosphors were dispersed in ethyl acetate at a weight ratio of 2.5:1 with mechanical stirring for 30 min. Subsequently, the dielectric-coated conductive fiber was dipped into the ZnS phosphor dispersion and then passed through a homemade die (with a diameter of 180 μm) at a speed of 10 m/min and dried at 80 °C to prepare the electroluminescent layer. A photo-conversion precursor was then prepared by mixing 50 *wt*.% WPU, $BaTiO_3$, and the GBP at a weight ratio of 1:2.75:0.5. The electroluminescent fiber was immersed in the precursor dispersion and processed under the same coating conditions to form the dielectric photo-conversion layer. In this layer, the GBP served as the photo-conversion component, enabling modulation of the emitted light through its photoluminescent behavior, while $BaTiO_3$ maintained the dielectric properties of the coating. Finally, a silver nanowire (AgNWs) dispersion with a concentration of 4 mg/mL was coated onto the outer surface of the fiber to form the transparent electrode, preparing the multilayer coaxial fiber device. For water resistance, a PU layer was then deposited on the electroluminescent fiber electrode by dip-coating.

2. Figures and Tables

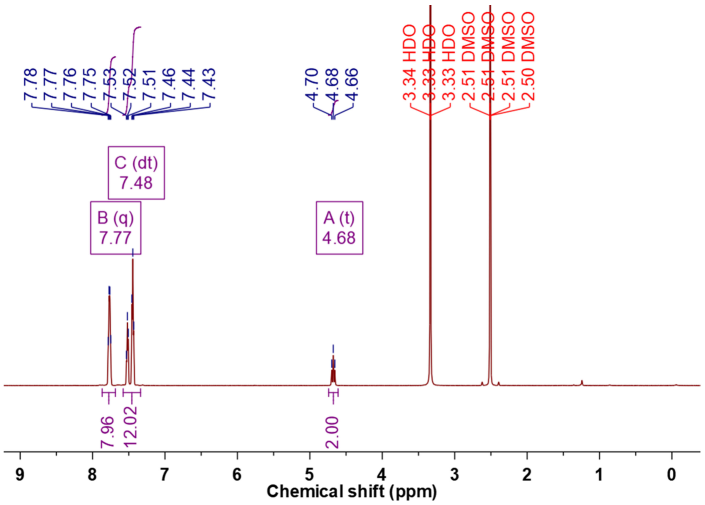

Figure S1. ^{1}H NMR spectrum of $Au_2(\mu\text{-DPPM})Cl_2$ in DMSO-d_6.

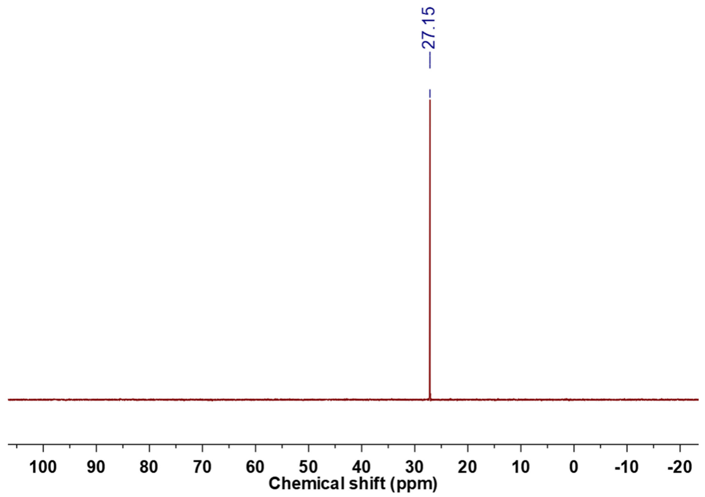

Figure S2. $^{31}P\{^{1}H\}$ NMR spectrum of $Au_2(\mu\text{-DPPM})Cl_2$ in DMSO-d_6.

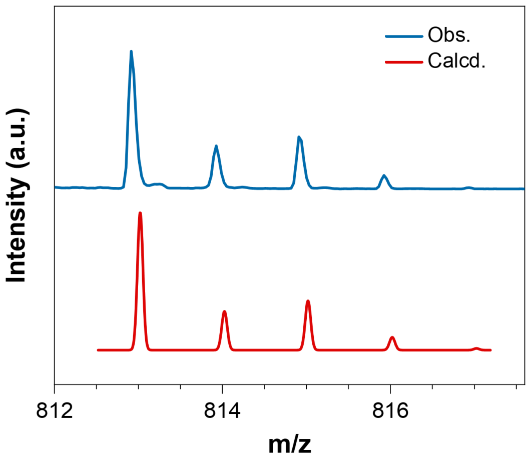

Figure S3. MALDI-TOF MS of Au_2(μ-DPPM)Cl_2.

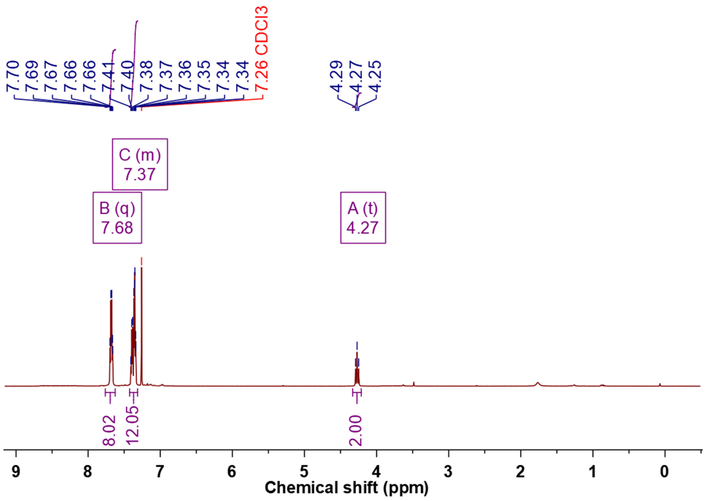

Figure S4. ^{1}H NMR spectrum of GBP in $CDCl_3$.

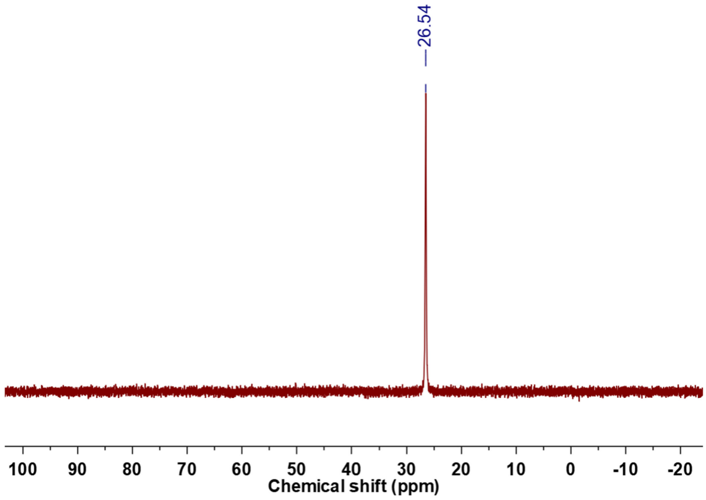

Figure S5. $^{31}P\{^{1}H\}$ NMR spectrum of GBP in $CDCl_3$.

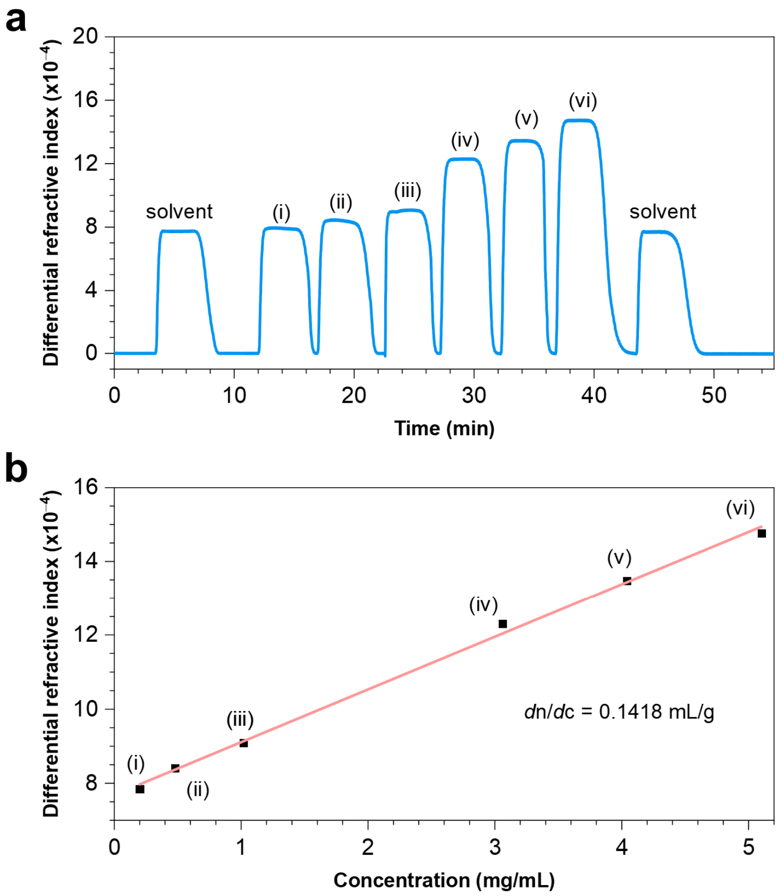

Figure S6. a) Overlay of differential refractive index signals recorded for GBP/DMF solutions at different concentrations. b) Linear fit of the peak height as a function of concentration, yielding a *d*n/*d*c value of 0.1418 mL/g for GBP in DMF. Concentrations: (i) 0.20, (ii) 0.48, (iii) 1.02, (iv) 3.06, (v) 4.04, and (vi) 5.10 mg/mL.

Table S1. Single crystal data of $[Au_3(\mu\text{-DPPM})_2Cl_2]ClO_4$.

	$[Au_3(\mu\text{-DPPM})_2Cl_2]ClO_4$
Formula	$C_{50}H_{44}Au_3Cl_2P_4$, ClO_4
Formula Weight	1529.98
Wavelength (Å)	1.54184
Space Group	P –1
	a = 16.1462(4)
Cell Length (Å)	b = 18.7091(2)
	c = 19.3138(6)
	α = 84.698(2)
Cell Angle (°)	β = 65.381(3)
	γ = 68.894(2)
Cell Volume (Å^3)	4936.8(3)
Z	4
Density (g/cm^3)	2.059
F (000)	2896.0
h_{max}, k_{max}, l_{max}	20, 22, 24
T_{min}, T_{max}	0.638, 1.000
R	0.0524(16766)
wR2	0.1110(19500)
Data completeness	0.948

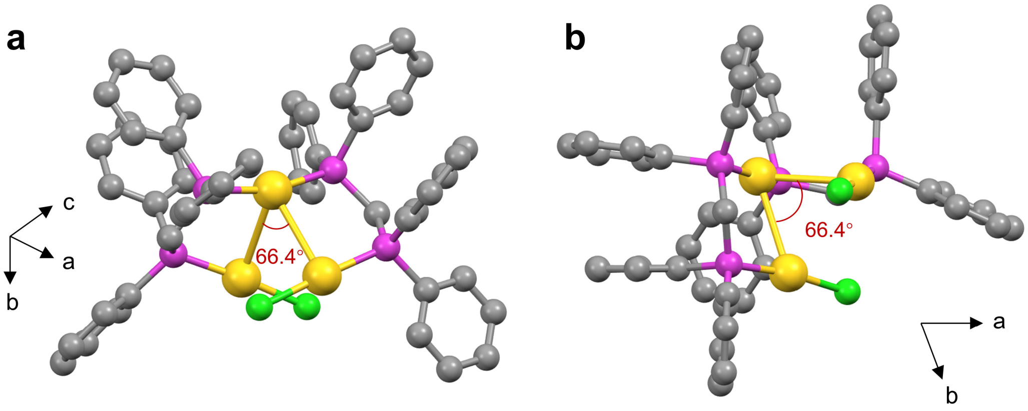

Figure S7. Single crystal structure of the chlorine-terminated trinuclear oligomer [Au_3(μ-DPPM)$_2Cl_2$]ClO_4, showing the bond angle (Au1–Au2–Au3) of 66.4°. For clarity, the counterions are omitted.

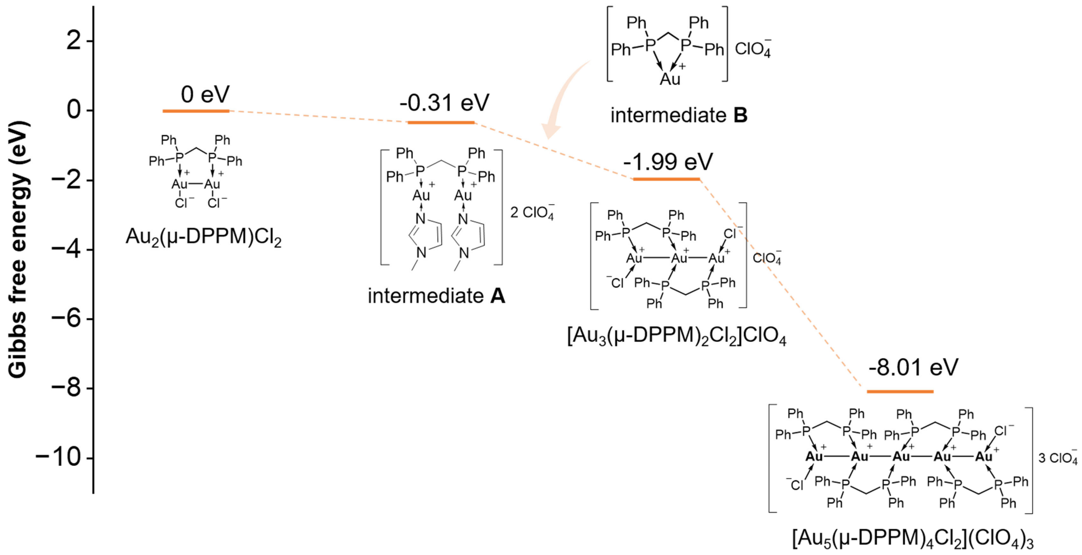

Figure S8. Gibbs free energy diagrams for the chain propagation pathways. The calculated free-energy profile shows a continuous decrease in Gibbs free energy upon successive Au-chain elongation, indicating that the growth process is thermodynamically favorable under the adopted computational model.

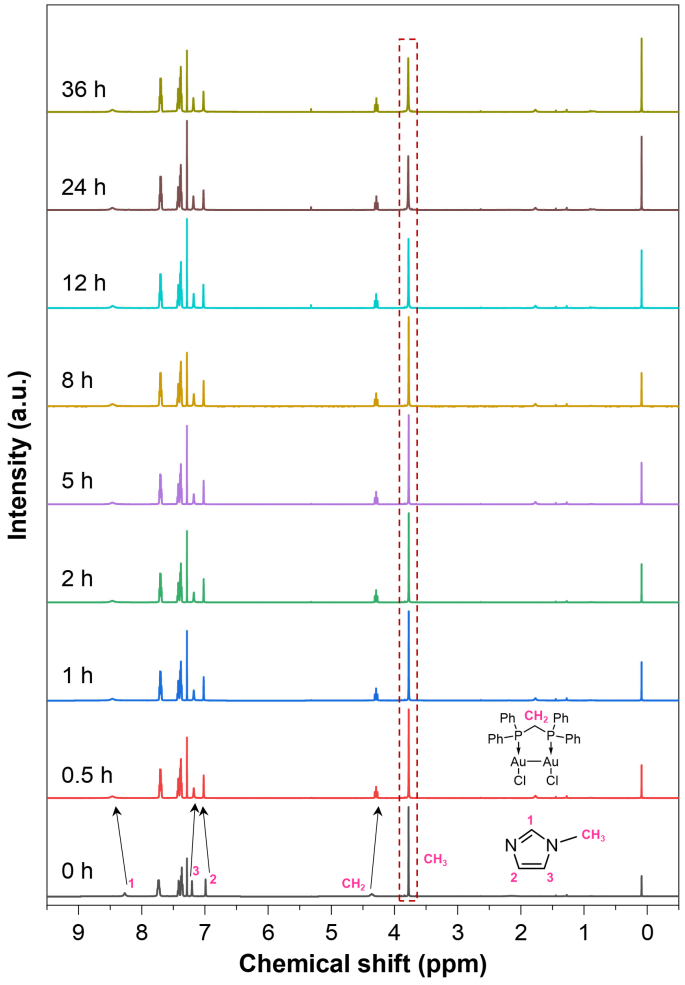

Figure S9. *In situ* ^{1}H NMR full spectra of the polymerization system.

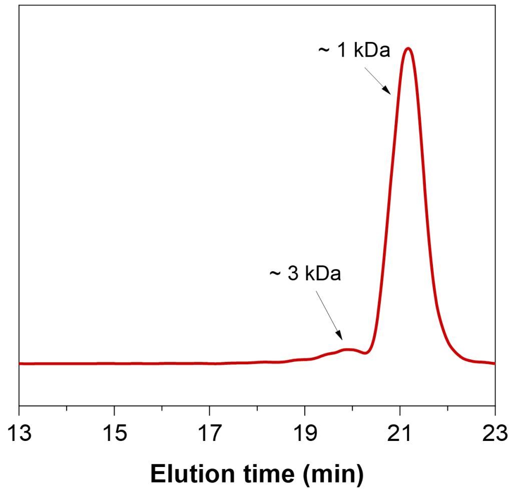

Figure S10. GPC trace of the crude product without adding N-MeIm.

Note S1. Without N-MeIm, only low-molecular-weight species from the crude product were detected (M_w < 3 kDa, Fig. S10), indicating that no efficient chain propagation occurs and highlighting the essential role of the N-MeIm in the polymerization.

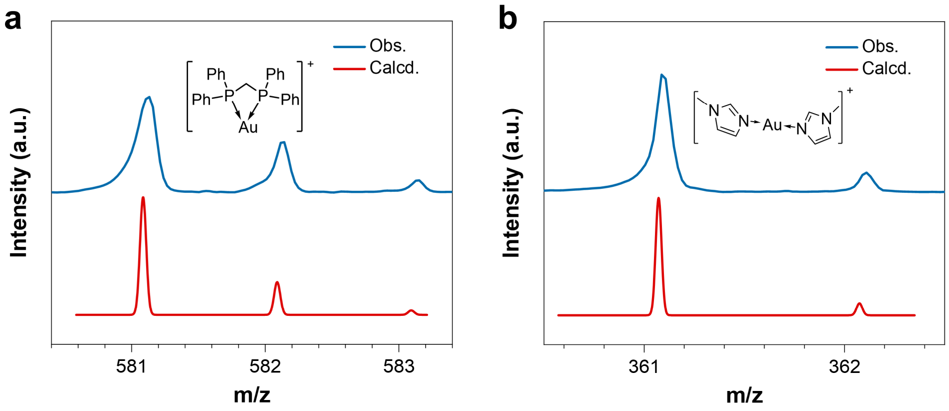

Figure S11. MALDI-TOF MS of the captured intermediate a) **B** and b) **C**.

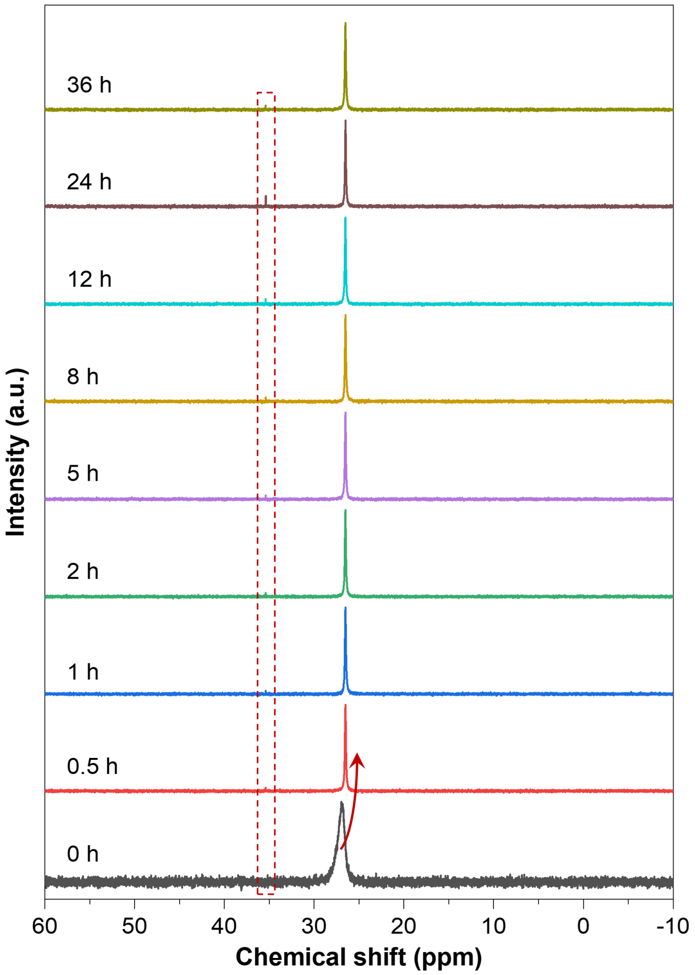

Figure S12. *In situ* $^{31}P\{^{1}H\}$ NMR full spectra of the polymerization system.

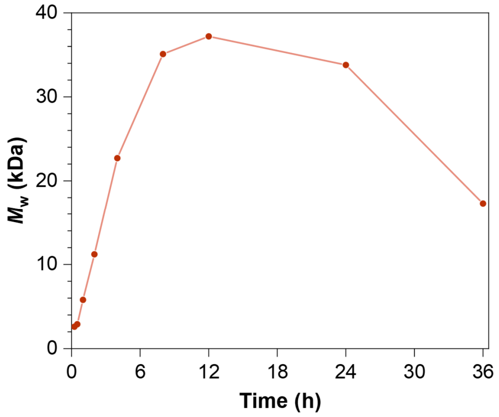

Figure S13. The evolution of M_w in the polymerization system.

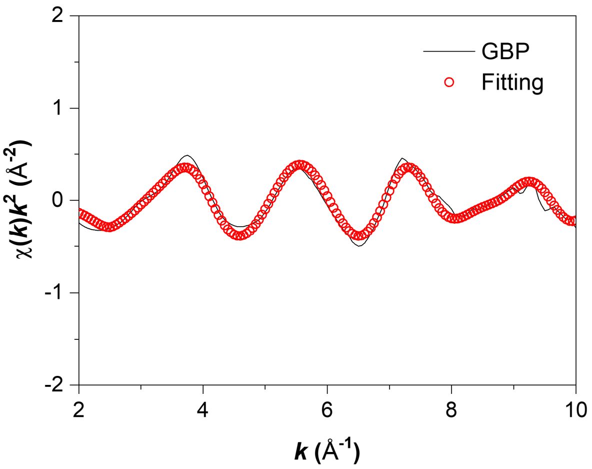

Figure S14. Au L_3-edge EXAFS (line) and curve fit (points) shown in *k*-space for GBP. The data are k^2-weighted and not phase-corrected.

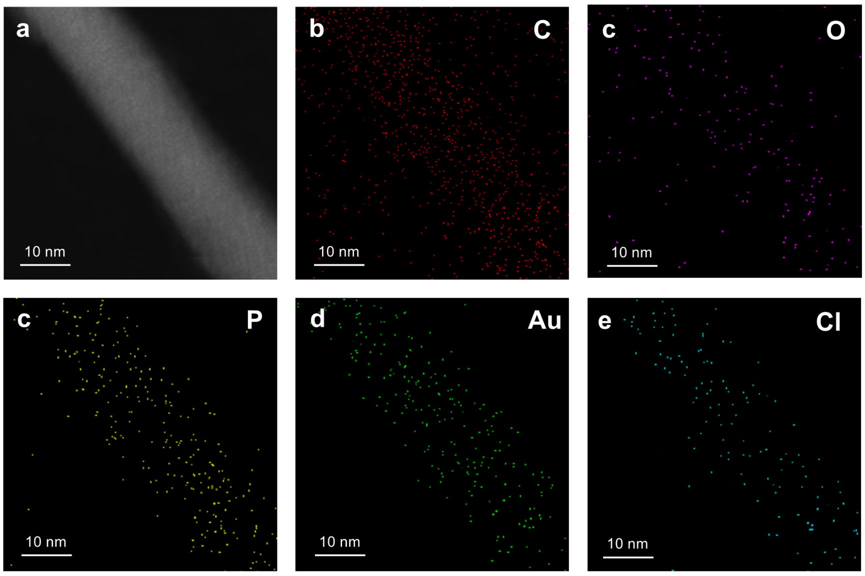

Figure S15. (a) Dark-field TEM image of GBP. (b–e) EDS elemental mapping of C, O, P, Au and Cl, respectively.

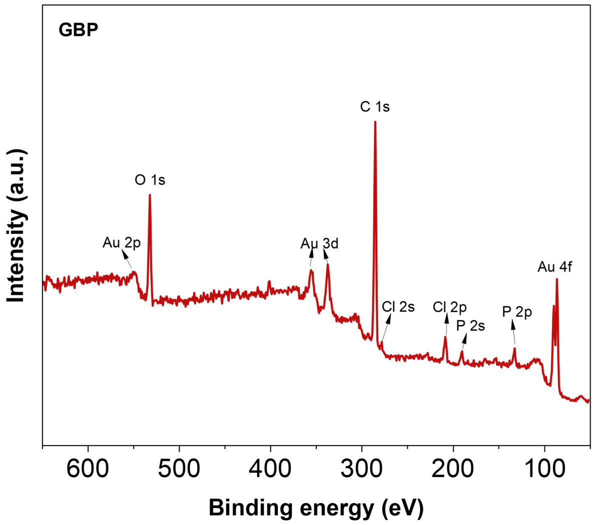

Figure. S16. XPS survey scan of GBP.

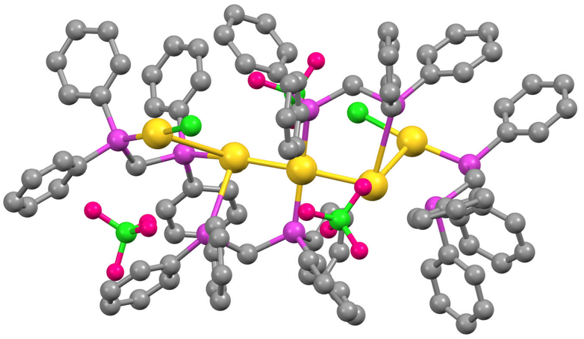

Figure S17. The complete density functional theory-optimized structure for the chlorine-terminated pentanuclear oligomer $[Au_5(\mu\text{-DPPM})_4Cl_2](ClO_4)_3$.

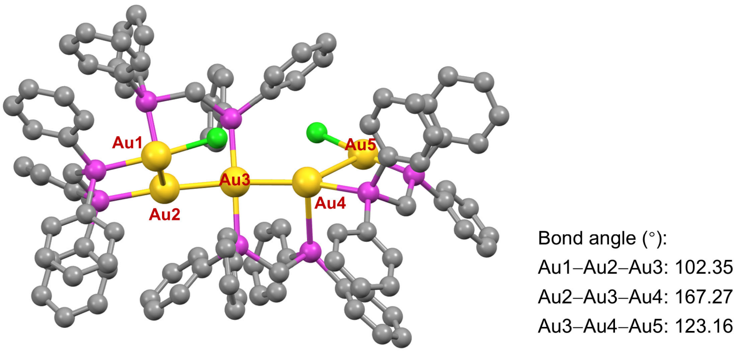

Figure S18. Density functional theory-optimized structure for the chlorine-terminated pentanuclear oligomer $[Au_5(\mu\text{-DPPM})_4Cl_2](ClO_4)_3$, showing the bond angles in the Au backbone. For clarity, the counterions are omitted.

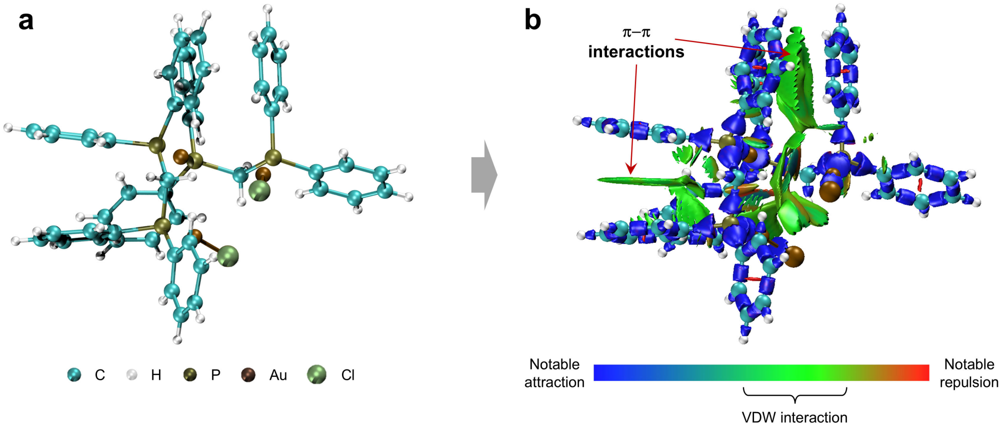

Figure S19. IRI analysis of $[Au_3(\mu\text{-DPPM})_2Cl_2]ClO_4$. a) Molecular structure. b) IRI plot of $[Au_3(\mu\text{-DPPM})_2Cl_2]ClO_4$, showing the π–π interactions among the adjacent phenyl rings. For clarity, the counterions are omitted. The red, green, and blue surfaces represent strong repulsion, van der Waals interaction, and strong attraction, respectively.

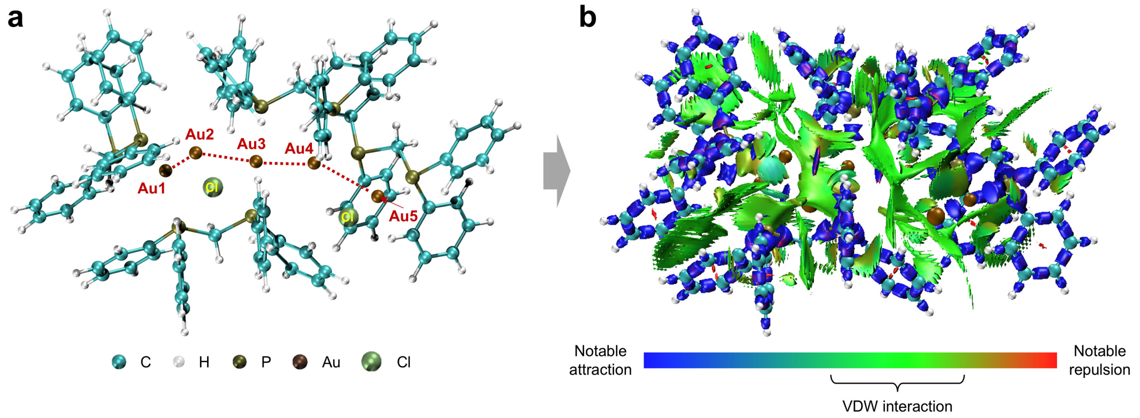

Figure S20. IRI analysis of $[Au_5(\mu\text{-DPPM})_4Cl_2](ClO_4)_3$. a) Molecular structure. b) IRI plot of $[Au_5(\mu\text{-DPPM})_4Cl_2](ClO_4)_3$, showing the π–π interactions among the adjacent phenyl rings. For clarity, the counterions are omitted. The red, green, and blue surfaces represent strong repulsion, van der Waals interaction, and strong attraction, respectively.

Table S2. EXAFS fitting parameters at the Au L_3-edge for various samples.

Sample	Shell	CN[a]	R(Å)[b]	σ^2(Å^2)[c]	ΔE_0(eV)[d]	R factor[e]
Au foil	Au-Au	12.0	2.86±0.01	0.0077	4.5	0.0023
$Au_2(\mu\text{-DPPM})Cl_2$	Au-P	1.0	2.28±0.01	0.0077	9.5	0.0093
	Au-Cl	1.0	2.23±0.01	0.0030	5.8	
GBP	Au-P	1.9±0.3	2.22±0.01	0.0051	-1.8	0.0189
	Au-Au	2.1±0.2	2.75±0.01	0.0080	11.2	

[a]CN, coordination number; [b]R, distance between absorber and backscatter atoms; [c]σ2, Debye-Waller factor to account for both thermal and structural disorders; [d]ΔE_0, inner potential correction; [e]R factor indicates the goodness of the fit. The amplitude reduction factor S_0^2 value was fixed to 0.850 (Au), according to the experimental EXAFS fit of Au foil by fixing CN as the known crystallographic value. A reasonable range of EXAFS fitting parameters: $0.700 < S_0^2 < 1.000$; CN > 0; $\sigma^2 > 0$; $|\Delta E_0| < 15$ eV; R factor < 0.02.

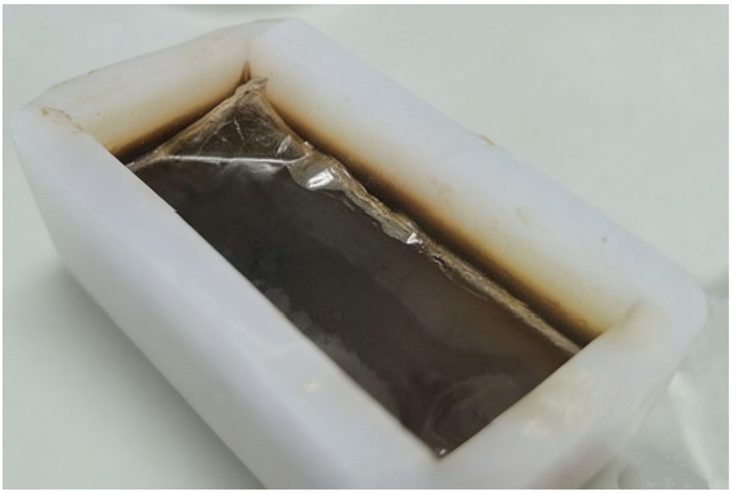

Figure S21. Photograph of a free-standing GBP film, showing the excellent film-forming ability of GBP. The film was prepared by solution casting from GBP/dichloromethane solution.

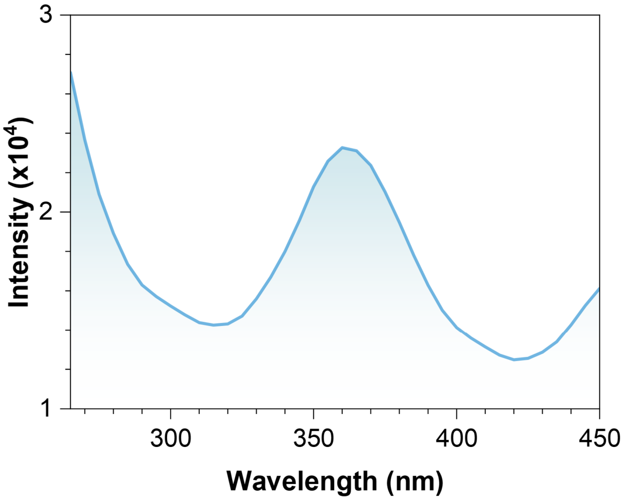

Figure S22. Excitation spectrum of GBP solid powder (emission wavelength = 490 nm).

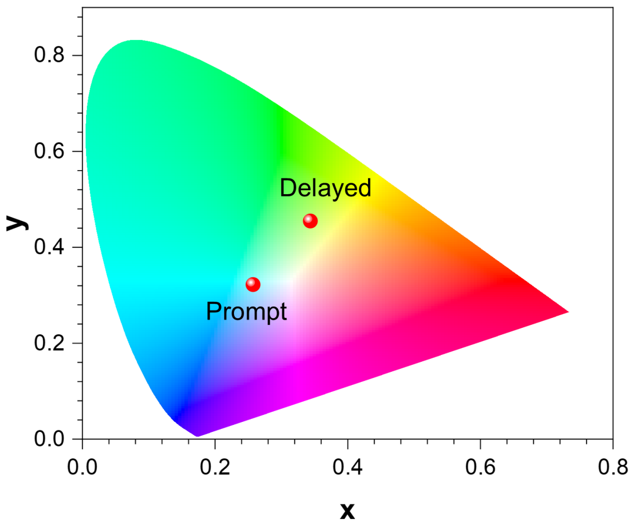

Figure S23. Commission Internationale de l'Éclairage coordinate diagram of prompt and delayed emission of GBP solid powders.

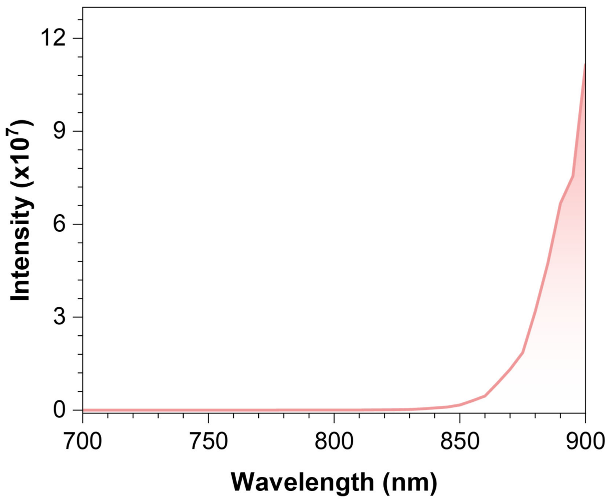

Figure S24. Delayed emission spectrum (t_d = 0.1 ms) of GBP solid powders in the 700–900 nm range (excitation wavelength = 365 nm).

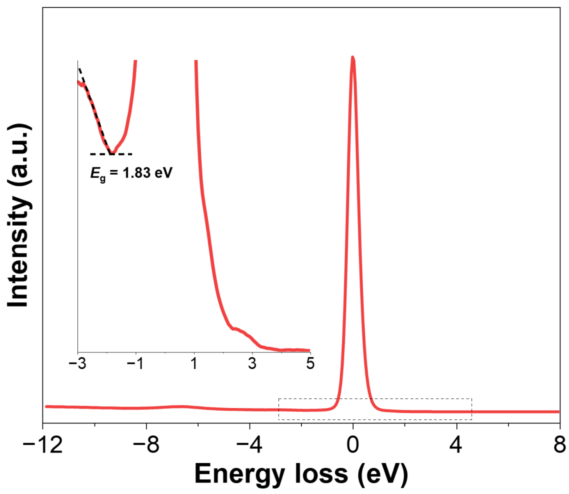

Figure S25. Reflection electron energy loss spectroscopy spectrum of GBP.

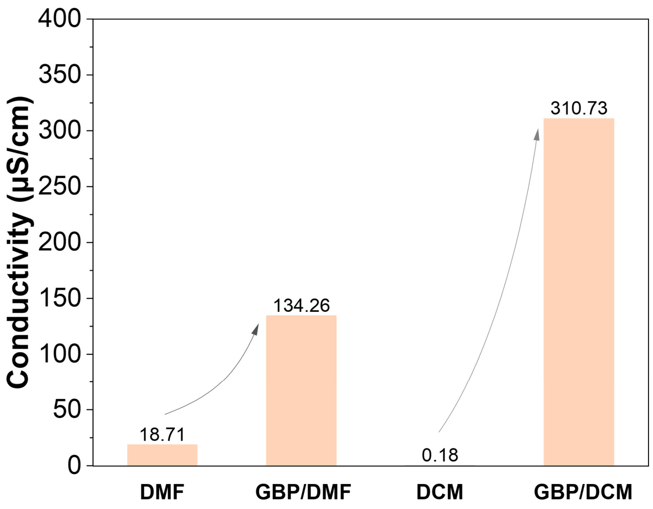

Figure S26. Comparison of electrical conductivities between 2 mg/mL GBP solutions in *N,N*-dimethylformamide and dichloromethane, and those of their corresponding pure solvents.

3. Cartesian Coordinate of the Optimized Structures for [Au_5(μ-DPPM)$_4$$Cl_2$]($ClO_4$)$_3$

Standard orientation:

Atomic	X	Y	Z
P	-6.22974200	0.29347900	-1.60394500
P	0.15250200	2.47857200	0.41148500
C	-6.51241500	0.21155000	0.26801100
P	-5.13357000	1.02618200	1.31477900
Au	-2.72713800	0.45585800	0.69720100
Au	-4.05050600	-0.32825400	-2.28445500
C	1.86287300	3.35278200	0.56429200
P	3.32953800	2.37596300	1.32120300
Au	-0.07515300	0.07443200	0.27314700
P	-0.25489700	-2.32540100	0.46353100
C	-1.88517800	-3.16313500	-0.07591500
P	-3.47170700	-2.80674900	0.92962300
Au	2.47609900	0.00988400	-0.65239600
P	6.79673700	-0.61631400	-0.34683100
C	6.14950700	0.74206400	-1.50134200
P	4.40886900	0.43946300	-2.22161400
Au	5.27299900	-1.45587800	1.24767900
C	-5.64187300	2.84596600	1.31411400
C	-6.66355600	3.33581300	2.14329800
C	-7.01692400	4.68951900	2.09679600
C	-6.35516800	5.55863200	1.22022900
C	-5.33258200	5.07370700	0.39603900
C	-4.96945100	3.72255400	0.44798600
C	-5.56060700	0.44560100	3.05391800
C	-4.55697300	0.57433800	4.03098100
C	-4.80004900	0.16805600	5.34709100
C	-6.04109900	-0.37794900	5.69864700
C	-7.03950500	-0.51650700	4.72855100
C	-6.80213800	-0.10525400	3.41009200
C	-7.57971100	-0.80195100	-2.32260400
C	-8.76945200	-1.07318900	-1.63044900
C	-9.76989700	-1.84504100	-2.23374500
C	-9.58841200	-2.34323500	-3.52880800
C	-8.39988800	-2.07642800	-4.21889400
C	-7.39448800	-1.31175100	-3.61766100

C	-6.71720500	2.02486800	-2.15109500
C	-5.84291000	2.75640800	-2.96763200
C	-6.21448100	4.02552400	-3.42930400
C	-7.45504600	4.56539800	-3.07466000
C	-8.32942500	3.83411500	-2.25968900
C	-7.96533100	2.56396700	-1.80280700
C	4.69968000	3.68877800	1.15378500
C	5.77337800	3.64862300	2.06595800
C	6.86254800	4.51466400	1.92541500
C	6.90155300	5.43529400	0.87044200
C	5.84574300	5.47663000	-0.04580600
C	4.75389600	4.60794500	0.09089900
C	3.00478900	2.44574400	3.18513400
C	2.85606000	3.65021100	3.89425800
C	2.64718900	3.63307900	5.27720000
C	2.58880400	2.41179000	5.96433400
C	2.74217800	1.20980300	5.26459200
C	2.95016000	1.22373600	3.87694200
C	-0.75406700	3.21163900	1.90048700
C	-1.63961500	4.29117100	1.77974700
C	-2.27967800	4.80635600	2.91523500
C	-2.03506600	4.25161900	4.17530000
C	-1.15086000	3.17148100	4.29728300
C	-0.51680800	2.64919400	3.16538700
C	-0.50336800	3.31595000	-1.14058400
C	-0.36257800	4.70157500	-1.33580100
C	-0.81498900	5.29480400	-2.52079900
C	-1.39299400	4.50696200	-3.52441900
C	-1.51823100	3.12436300	-3.33977500
C	-1.08002400	2.52913000	-2.14911000
C	-3.38535000	-4.08765400	2.33148800
C	-3.46918400	-3.62009200	3.65181300
C	-3.45230800	-4.52209100	4.72433200
C	-3.35434600	-5.89641400	4.48410400
C	-3.27575200	-6.37011000	3.16709400
C	-3.29312500	-5.47022700	2.09610800
C	-4.75692000	-3.67827800	-0.17787700
C	-5.99993100	-3.98416600	0.41046900
C	-7.01803700	-4.58362200	-0.33740800
C	-6.81300900	-4.88518200	-1.68970000
C	-5.58680500	-4.57732800	-2.28519300

C	-4.56357500	-3.97678900	-1.53590600
C	0.00008800	-2.79326400	2.26250400
C	0.09870300	-4.14011600	2.64547600
C	0.28523600	-4.47254400	3.99031600
C	0.37029000	-3.46548700	4.95889000
C	0.26556600	-2.12350600	4.58047900
C	0.08211200	-1.78740300	3.23461600
C	0.94522700	-3.42170400	-0.50204700
C	2.11641100	-3.90760900	0.09974800
C	2.95920600	-4.77088800	-0.61293600
C	2.64608200	-5.14450000	-1.92586100
C	1.49484400	-4.63048600	-2.53654400
C	0.64735600	-3.76627100	-1.83152600
Cl	3.97437000	-2.38871900	3.01229700
Cl	-1.97234100	-1.05621700	-3.24849900
C	4.08922800	2.05984100	-3.13999100
C	2.78353500	2.27348000	-3.61781000
C	2.47568900	3.43698300	-4.33122500
C	3.46480100	4.40025000	-4.56787900
C	4.76238500	4.19584300	-4.08787800
C	5.07590800	3.02949900	-3.37645000
C	4.69281100	-0.77189600	-3.63698400
C	3.90502800	-1.92975700	-3.69655400
C	4.06740500	-2.82818100	-4.75937900
C	5.01187700	-2.57410100	-5.75856600
C	5.79682600	-1.41494100	-5.70091800
C	5.63440600	-0.51297300	-4.64572200
C	7.51564600	-1.93835400	-1.47309800
C	6.82354400	-3.14706800	-1.63895900
C	7.34645300	-4.14015600	-2.47576700
C	8.56001800	-3.93156000	-3.14011800
C	9.25410300	-2.72684100	-2.96895200
C	8.73541100	-1.72984900	-2.13604600
C	8.28533300	0.17396500	0.48517500
C	8.74344600	-0.39434300	1.68578700
H	-7.47829500	0.65984400	0.51499500
H	-6.52226700	-0.85226700	0.52156400
H	2.15250300	3.56410800	-0.46765800
H	1.70012700	4.29434100	1.09676900
H	-1.68942200	-4.23794400	-0.12236500
H	-2.06240100	-2.78361500	-1.08508700

C	9.86703100	0.13618600	2.32807600
C	10.53445300	1.23863500	1.78098500
H	-7.17315900	2.67238100	2.83469900
H	-7.80381900	5.06361200	2.74431200
H	-6.63300000	6.60732900	1.18325400
H	-4.81720600	5.74295900	-0.28445000
H	-4.16298400	3.35112900	-0.17588400
H	-3.58727500	0.98294700	3.75926500
H	-4.02051600	0.27250400	6.09443000
H	-6.22718300	-0.69507800	6.71946700
H	-8.00283800	-0.94018000	4.99425400
H	-7.59202100	-0.21908800	2.67474200
H	-8.92742800	-0.69700700	-0.62559600
H	-10.68735100	-2.05298800	-1.69291600
H	-10.36690000	-2.93771300	-3.99563700
H	-8.25119500	-2.46630400	-5.22029800
H	-6.46682100	-1.12143200	-4.14883700
H	-4.87891500	2.33741400	-3.23922700
H	-5.53661000	4.58586300	-4.06480200
H	-7.74369400	5.54750500	-3.43441300
H	-9.29423400	4.24810200	-1.98681000
H	-8.66109400	2.00013500	-1.18934900
H	5.75363800	2.94771600	2.89446200
H	7.67613700	4.47257600	2.64267800
H	7.74271400	6.11324700	0.76687300
H	5.86223900	6.18831900	-0.86565700
H	3.95388500	4.66303000	-0.63876600
H	2.91903200	4.60195900	3.37530700
H	2.53765100	4.56761500	5.81863600
H	2.43889400	2.40152100	7.03943600
H	2.72027100	0.25992800	5.78894600
H	3.08723000	0.28570900	3.34774300
H	-1.85341500	4.72178600	0.80925900
H	-2.97517900	5.63183100	2.80728300
H	-2.52813600	4.65522700	5.05369400
H	-0.94542700	2.73921800	5.27098000
H	0.16229100	1.81171400	3.27566400
H	0.10020800	5.32278200	-0.57586100
H	-0.70819200	6.36580700	-2.66006800
H	-1.73423700	4.96773100	-4.44645200
H	-1.94392600	2.49784300	-4.11687000

H	-1.18359800	1.45714400	-2.01885100
H	-3.54845000	-2.55408200	3.84056400
H	-3.51723500	-4.15037800	5.74186800
H	-3.34629700	-6.59582800	5.31441500
H	-3.21003200	-7.43731400	2.97540200
H	-3.25656600	-5.84910900	1.07860000
H	-6.16251900	-3.78063200	1.46683700
H	-7.96405200	-4.82658000	0.13866900
H	-7.60105700	-5.35320600	-2.27027000
H	-5.41558300	-4.80538400	-3.33404700
H	-3.62674900	-3.75002800	-2.02924100
H	0.03936500	-4.92971500	1.90523800
H	0.36321700	-5.51525800	4.27917800
H	0.52423100	-3.72729700	6.00090600
H	0.34239400	-1.33764100	5.32415500
H	0.00760400	-0.74456100	2.93841100
H	2.38149300	-3.62226500	1.11180300
H	3.85077300	-5.15807600	-0.12928900
H	3.28964000	-5.82831700	-2.46688200
H	1.24621700	-4.90560100	-3.55658400
H	-0.22899000	-3.36255000	-2.32747300
C	10.07532300	1.81068700	0.58987900
C	8.95429300	1.27977700	-0.05958800
H	6.87253400	0.88329900	-2.30905400
H	6.08338300	1.65512000	-0.90247100
H	2.00885000	1.53759800	-3.42619100
H	1.46441700	3.59056900	-4.69138700
H	3.22550200	5.30225000	-5.12193000
H	5.53415200	4.93738200	-4.26844400
H	6.08813500	2.89595000	-3.01237500
H	3.16729100	-2.12659500	-2.92495800
H	3.45221200	-3.71974500	-4.80207600
H	5.13422600	-3.26957200	-6.58248400
H	6.52476700	-1.21097200	-6.47901700
H	6.22876900	0.39516000	-4.62610300
H	5.88622400	-3.31346100	-1.11771000
H	6.80890400	-5.07391600	-2.60223900
H	8.96672700	-4.70573100	-3.78204700
H	10.20053400	-2.56653500	-3.47389800
H	9.29212700	-0.80924400	-1.98959800
H	8.21903500	-1.23869800	2.12247100

H	10.21369000	-0.30747100	3.25527200
H	11.40381500	1.65056400	2.28188300
H	10.58654600	2.66835800	0.16410000
H	8.61012300	1.74073500	-0.97984600
Cl	0.26007349	-1.36807732	-2.67138494
O	0.33459983	-0.07592023	-3.41178884
O	-0.47737848	-2.38326081	-3.50817313
O	1.67799065	-1.88565281	-2.48571525
O	-0.39187732	-1.20818520	-1.35465014
Cl	-1.73589723	0.16939218	5.74537347
O	-2.55914081	-1.00535649	5.33836286
O	-2.53050886	1.03454537	6.69128558
O	-0.53009894	-0.34608129	6.51555806
O	-1.28629742	0.95459423	4.57673595
Cl	6.23897635	1.50724275	4.10372025
O	7.50231076	0.72173473	4.00181160
O	5.38039626	0.93488274	5.20367099
O	6.60025316	2.92740046	4.51050995
O	5.50002065	1.52478254	2.82386373

4. References

[1] A. D. Becke, *J. Chem. Phys.* **1993**, *98*, 5648.

[2] C. Lee, W. Yang, R. G. Parr, *Phys. Rev. B* **1988**, *37*, 785–789.

[3] W. J. Hehre, R. Ditchfield, J. A. Pople, *J. Chem. Phys.* **1972**, *56*, 2257–2261.

[4] P. J. Hay, W. R. Wadt, *J. Chem. Phys.* **1985**, *82*, 270–283.

[5] M. J. Frisch, G. W. Trucks, H. B. Schlegel, G. E. Scuseria, M. A. Robb, J. R. Cheeseman, G. Scalmani, V. Barone, G. A. Petersson, H. Nakatsuji, X. Li, M. Caricato, A. V. Marenich, J. Bloino, B. G. Janesko, R. Gomperts, B. Mennucci, H. P. Hratchian, J. V. Ortiz, A. F. Izmaylov, J. L. Sonnenberg, D. Williams-Young, F. Ding, F. Lipparini, F. Egidi, J. Goings, B. Peng, A. Petrone, T. Henderson, D. Ranasinghe, V. G. Zakrzewski, J. Gao, N. Rega, G. Zheng, W. Liang, M. Hada, M. Ehara, K. Toyota, R. Fukuda, J. Hasegawa, M. Ishida, T. Nakajima, Y. Honda, O. Kitao, H. Nakai, T. Vreven, K. Throssell, J. A. Montgomery, Jr., J. E. Peralta, F. Ogliaro, M. J. Bearpark, J. J. Heyd, E. N. Brothers, K. N. Kudin, V. N. Staroverov, T. A. Keith, R. Kobayashi, J. Normand, K. Raghavachari, A. P. Rendell, J. C. Burant, S. S. Iyengar, J. Tomasi, M. Cossi, J. M. Millam, M. Klene, C. Adamo, R. Cammi, J. W. Ochterski, R. L. Martin, K. Morokuma, O. Farkas, J. B. Foresman, and D. J. Fox, Gaussian, Inc. Wallingford CT, **2016**.

[6] T. Lu, F. Chen, *J. Comput. Chem.* **2012**, *33*, 580–592.

[7] F. Neese, *Wires. Comput. Mol. Sci.* **2012**, *2*, 73–78.

[8] F. Neese, *Wires. Comput. Mol. Sci.* **2025**, *15*, e70019.

[9] S. Grimme, A. Hansen, S. Ehlert, J.-M. Mewes, *J. Chem. Phy.* **2021**, 154.

[10] M. Cossi, N. Rega, G. Scalmani, V. Barone, *J. Comput. Chem.* **2003**, *24*, 669–681.

[11] M.-C. Brandys, M. C. Jennings, R. J. Puddephatt, *J. Chem. Soc. Dalton Trans.* **2000**, 4601–4606.
